# NMDA Receptors Coordinate Metabolic Reprogramming and Mitophagy in Schwann Cells to Promote Peripheral Nerve Regeneration

**DOI:** 10.34133/research.0825

**Published:** 2025-08-05

**Authors:** Fangzhen Shan, Xiaoying Yao, Qingqing Zhang, Ruolin Li, Lingmeng Kong, Qian Zheng, Nannan Zhang, Yuzhong Wang

**Affiliations:** ^1^ Department of Neurology, Affiliated Hospital of Jining Medical University, Jining, Shandong 272029, China.; ^2^ Medical Research Centre, Affiliated Hospital of Jining Medical University, Jining, Shandong 272029, China.; ^3^ Key Laboratory of Cell and Biomedical Technology of Shandong Province, Jining, Shandong 272029, China.; ^4^ Cheeloo College of Medicine, Shandong University, Jinan, Shandong 250063, China.; ^5^ Department of Respiratory and Critical Care, Affiliated Hospital of Jining Medical University, Jining, Shandong 272029, China.

## Abstract

Schwann cells (SCs) are indispensable for peripheral nerve regeneration, yet the mechanisms enabling their metabolic adaptation to meet the energetic demands of axonal repair remain elusive. Here, we identify *N*-methyl-D-aspartate (NMDA) receptors as central regulators of SC metabolic plasticity. In a mouse model of acute motor axonal neuropathy, nerve injury led to a marked decrease in NMDA receptor expression on SC. Functional studies revealed that NMDA receptors mediate calcium influx to drive glycolysis and oxidative phosphorylation via the phosphoinositide 3-kinase/protein kinase B/mammalian target of rapamycin and hypoxia-inducible factor-1α/c-Myc pathways, ensuring adenosine triphosphate production for axonal repair. Simultaneously, NMDA receptors orchestrate ataxia telangiectasia-mutated and Rad3-related protein–autophagy related 13-dependent mitophagy to clear reactive oxygen species-damaged mitochondria, maintaining metabolic efficiency during energy stress. Targeted metabolomics, Seahorse flux, and molecular pathological analysis revealed NMDA receptor-dependent remodeling of glucose metabolism, tricarboxylic acid cycle, nucleotide synthesis, and mitochondrial ultrastructure in SC. NMDA receptor deficiency disrupts energy metabolism and impairs axonal survival following sciatic nerve injury, resulting in aggravated neurological deficits and hindered nerve regeneration. Crucially, NMDA receptor activation rescued axonal integrity and motor function in mice with acute motor axonal neuropathy, underscoring their therapeutic potential. Our findings establish NMDA receptors as dual regulators of SC energy metabolism and mitochondrial quality control, providing a novel strategy to enhance glia-axonal metabolic coupling in peripheral neuropathies.

## Introduction

Schwann cells (SCs), the principal glial cells of the peripheral nervous system (PNS), are indispensable for maintaining axonal integrity and facilitating nerve regeneration following injury [1]. Beyond their canonical role in myelination, SCs support neuronal metabolism through glia–axonal metabolic coupling, supplying energy substrates such as lactate and pyruvate to sustain neuronal adenosine triphosphate (ATP) production [2,3]. This metabolic interdependence becomes pivotal after nerve damage, as SCs undergo a glycolytic shift regulated by the adenosine monophosphate-activated protein kinase (AMPK)/mammalian target of rapamycin (mTOR) pathway to meet the heightened energy demands of axonal repair [2]. Disruptions in this metabolic adaptation, whether due to impaired glycolysis or mitochondrial dysfunction, result in axonal degeneration and functional deficits [4,5]. While recent studies underscore the importance of SC metabolic plasticity in nerve repair, the molecular mechanisms orchestrating this process remain poorly defined.

*N*-methyl-D-aspartate (NMDA) receptor is a heterotetrameric complex typically composed of 2 obligatory NR1 subunits and 2 regulatory NR2 (A to D) or NR3 subunits, known for their roles in synaptic transmission and plasticity [6,7]. In neurons, dysregulated NMDA receptor signaling contributes to neurodegenerative disorders such as Alzheimer’s disease and epilepsy [8,9]. These receptors are also expressed in glial cells, where they influence neuronal activity and synaptic plasticity by modulating calcium signaling and releasing neuroactive substances [10–12]. Recent evidence, however, implicates NMDA receptors in cellular metabolic regulation. Their activation in oligodendrocytes enhances glucose uptake and supports neuronal energy homeostasis [13,14]. In SCs, NMDA receptors have been implicated in pain hypersensitivity and Remak bundle organization [15], but their role in metabolic adaptation during nerve repair remains underexplored. Despite growing evidence of NMDA receptors’ metabolic functions in neural cells, their contribution to SC metabolic reprogramming and mitochondrial quality control in peripheral nerve regeneration has not been explored.

In this study, we investigated the role of NMDA receptors in SCs using a mouse model of acute motor axonal neuropathy (AMAN), a subtype of Guillain–Barré syndrome (GBS). We demonstrate that NMDA receptors act as metabolic hubs in SCs, driving glycolysis and oxidative phosphorylation through phosphoinositide 3 kinase (PI3K)/protein kinase B (AKT)/mTOR and hypoxia-inducible factor-1α (HIF-1α)/c-Myc pathways while simultaneously activating ataxia telangiectasia-mutated and Rad3-related protein (ATR)–autophagy related 13 (Atg13)-mediated mitophagy to clear damaged mitochondria. NMDA receptor deficiency disrupted energy metabolism, exacerbated axonal degeneration, and impaired functional recovery, whereas pharmacological activation restored metabolic flux and accelerated regeneration. These findings establish NMDA receptors as dual regulators of SC energy metabolism and mitochondrial quality control, offering a novel therapeutic strategy to enhance glia-axonal coupling in peripheral neuropathies.

## Results

### Peripheral neuropathy reduces NMDA receptor expression on SCs

**Fig. 1.**
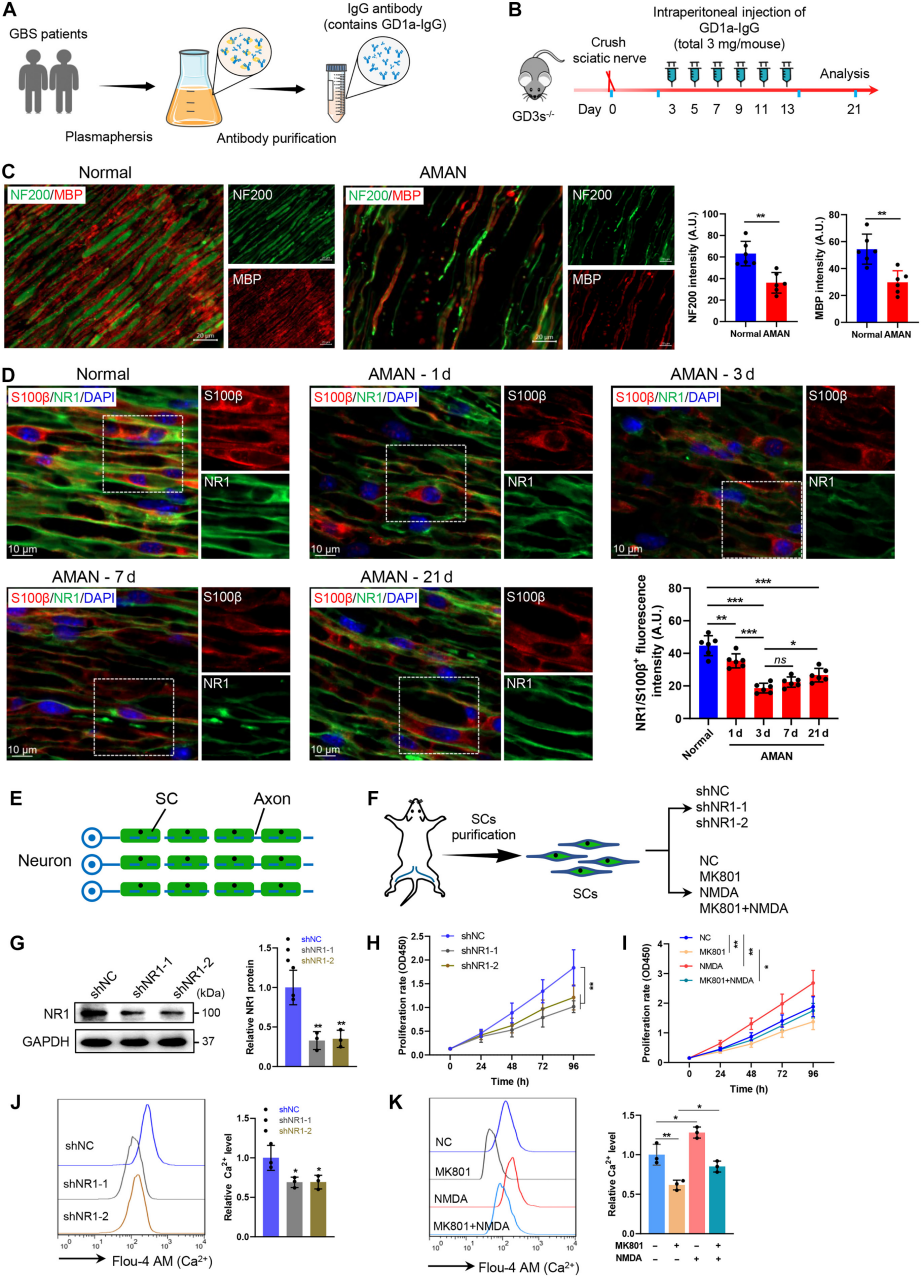
The expression of NMDA receptors was diminished in the lesion area of the AMAN mouse model. (A) Schematic diagram of GD1a-IgG purification. (B) Schematic diagram of the experimental process in the mouse model of AMAN. (C) Representative immunofluorescence and quantification of NF20 and MBP in the sciatic nerves of normal and AMAN mice. NF200 indicates axons, and MBP indicates myelin. *N* = 6, *n* ≥ 6 fields/group. Scale bar, 20 μm. (D) Representative immunofluorescence and quantification of NR1 in the sciatic nerves of normal and AMAN mice on days 1, 3, 7, and 21 after injury. S100β indicates SCs. The fluorescence intensity of NR1 in the S100β-positive region was statistically analyzed. Scale bar, 10 μm. *N* = 6, *n* ≥ 6 fields/group. (E) Schematic diagram of the sciatic nerve in mice. (F) Grouping scheme of purified SCs from neonatal C57BL/6J mouse nerves. MK801: 5 μM; NMDA: 100 μM. (G) The efficiency of NR1 knockdown in primary SCs using NR1-specific shRNAs was identified by Western blot. *n* = 3. (H and I) Cell proliferation was determined by a CCK-8 assay. *n* = 4. (J and K) Cytosolic Ca^2+^ was stained with Fluo-4AM, and the concentration was determined by flow cytometry. Panel (K) depicts the levels of cytosolic Ca^2+^ following treatment with NMDA for 10 min, MK801 for 30 min, or MK801 for 30 min followed by NMDA for 10 min. *n* = 3. *ns*, not significant, *P < 0.05, **P < 0.01, ***P < 0.001.

To investigate the effects of peripheral nerve injury on NMDA receptors, we analyzed sural nerve biopsies from 15 patients with immune peripheral neuropathy and 12 surgical amputation controls. Quantitative immunofluorescence analysis revealed a 40% reduction in NR1 expression on SCs in neuropathy samples compared to controls (*P* < 0.001; Fig. S1A). To further explore the role of NMDA receptors in peripheral neuropathy, we established a mouse model of AMAN by crushing sciatic nerve and administering intraperitoneal injection of anti-GD1a immunoglobulin G (IgG) antibody in GD3s^−/−^ mice (Fig. 1A and B). Histological analysis of distal sciatic nerve segments revealed severe axonal degeneration accompanied by myelin loss at the lesion site (Fig. 1C). NR1 expression in sciatic nerves of AMAN model mice was markedly decreased compared to controls (Fig. S1B). Quantitative reverse transcription polymerase chain reaction (qRT-PCR) analysis of NMDA receptor subunit expression in SCs detected relatively high expression of NR1, NR2C, and NR2D, with low expression of NR2A, NR2B, NR3A, and NR3B (Fig. S1C). Immunofluorescence staining showed that in the AMAN mice, NR1 expression on SCs was markedly decreased at 24 h postinjury, reached its nadir at 72 h, partially recovered at 3 weeks postinjury but remained below normal levels (Fig. 1D). NR2C/D expression on SCs within the lesion area decreased synchronously with NR1 (Fig. S1D), suggesting coordinated suppression of functional receptor complexes. In contrast, NR1 expression on neurons within the lesion area showed no notable alteration in AMAN model mice (Fig. S2A). Furthermore, immunofluorescence staining revealed markedly increased NR1 expression in macrophages at day 3 postinjury in AMAN model mice, which gradually decreased and returned to baseline levels by day 21 (Fig. S2B).

### NMDA receptor deficiency impairs SC proliferation

SC proliferation is critical for peripheral nerve repair, enabling remyelination and metabolic support to regenerating axons [2,16] (Fig. 1E). To investigate NMDA receptor function in this process, primary SCs were isolated from neonatal mice and subjected to NR1 subunit knockdown via 2 independent short hairpin RNAs (shRNAs) (Fig. 1F and G and Fig. S3). Pharmacological inhibition (MK801) or genetic silencing NR1 markedly reduced SC proliferation, while receptor activation (NMDA) enhanced proliferation (Fig. 1H and I). Cotreatment with NMDA rescued MK801-induced proliferative deficits (Fig. 1I), confirming the specificity of NMDA receptor signaling.

Calcium influx through NMDA receptors is a key regulator of cellular proliferation and metabolic adaptation [13,17]. In SCs, NR1 knockdown or MK801 treatment diminished intracellular Ca^2+^ levels (Fig. 1J and K), correlating with impaired proliferation. Conversely, NMDA-induced receptor activation restored Ca^2+^ influx and normalized proliferation (Fig. 1K). These findings establish NMDA receptor-mediated calcium signaling as a critical driver of SC proliferation during nerve repair.

### NMDA receptor deficiency disrupts energy-related metabolic pathways in SCs

Energy metabolism is critical for SC proliferation, dedifferentiation, and redifferentiation. To evaluate how NMDA receptor deficiency affects SC energy metabolism, we performed targeted metabolomic profiling on control and NR1-deficient SCs. Cluster analysis, principal component analysis, and partial least squares-discriminant analysis of 50 identified metabolites showed distinct metabolic clustering between NR1-deficient and control groups (Fig. S4), indicating profound metabolic reprogramming.

**Fig. 2.**
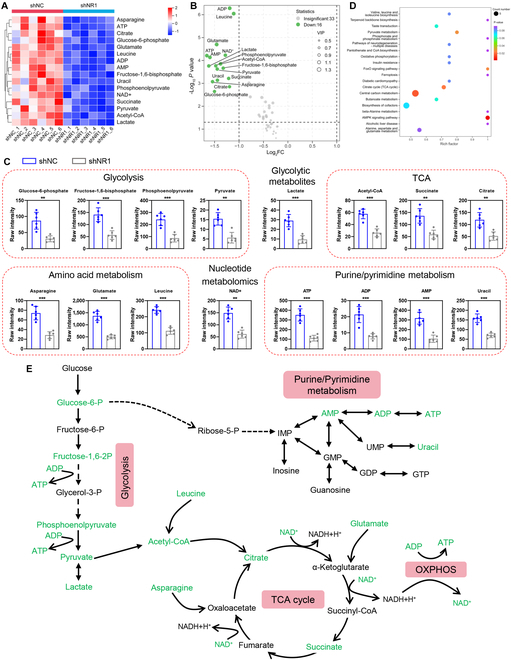
Metabolomic analysis of changes in SCs energy metabolism caused by NR1 deficiency. (A) Heatmap of differentially enriched metabolites caused by NR1 deficiency in energy metabolomics analysis. (B) Volcano map of the differentially enriched metabolites. Blue plots represent down-regulated metabolites in the NR1-deficient group compared to the control group. (C) Quantification of the differentially enriched metabolites. *n* = 6. (D) KEGG enrichment pathway analysis based on the differentially enriched metabolites. (E) Summary of metabolic pathways and targets impacted by NR1 deficiency in SCs. Schematic diagram of the metabolic pathways associated with glycolysis, TCA cycle, oxidative phosphorylation, and purine/pyrimidine metabolism. Metabolites that were markedly reduced in NR1-deficient SCs compared to controls are highlighted in green. **P < 0.01, ***P < 0.001.

Cluster heatmap and volcano plot highlighted the notable reductions in glycolytic metabolites (glucose 6-phosphate, fructose-1,6-bisphosphate, phosphoenolpyruvate, pyruvate, and lactate), tricarboxylic acid (TCA) cycle intermediates (acetyl-CoA, succinate, and citrate), and oxidative phosphorylation components (adenosine triphosphate (ATP) and nicotinamide adenine dinucleotide (NAD^+^)) in NR1-deficient SCs (Fig. 2A to C). Concurrently, amino acids critical for protein synthesis (arginine, glutamate, and leucine) and purine/pyrimidine metabolites (uracil, adenosine monophosphate [AMP], and adenosine diphosphate [ADP]) were markedly reduced (Fig. 2A to C), reflecting broad impairments in nucleotide and energy metabolism.

Kyoto Encyclopedia of Genes and Genomes (KEGG) pathway analysis implicated NMDA receptor deficiency in the dysregulation of AMPK signaling, FoxO signaling, central carbon metabolism, pyruvate metabolism, and neurodegenerative pathways (Fig. 2D). Enrichment analysis further confirmed substantial disruptions in glycolysis, TCA cycle activity, oxidative phosphorylation, amino acid metabolism, and nucleotide synthesis (Fig. 2E). Collectively, these findings underscore the pivotal role of NMDA receptors in the energy metabolism pathways of SCs, which are vital for peripheral nerve regeneration.

### NMDA receptors modulate glycolytic flux

**Fig. 3.**
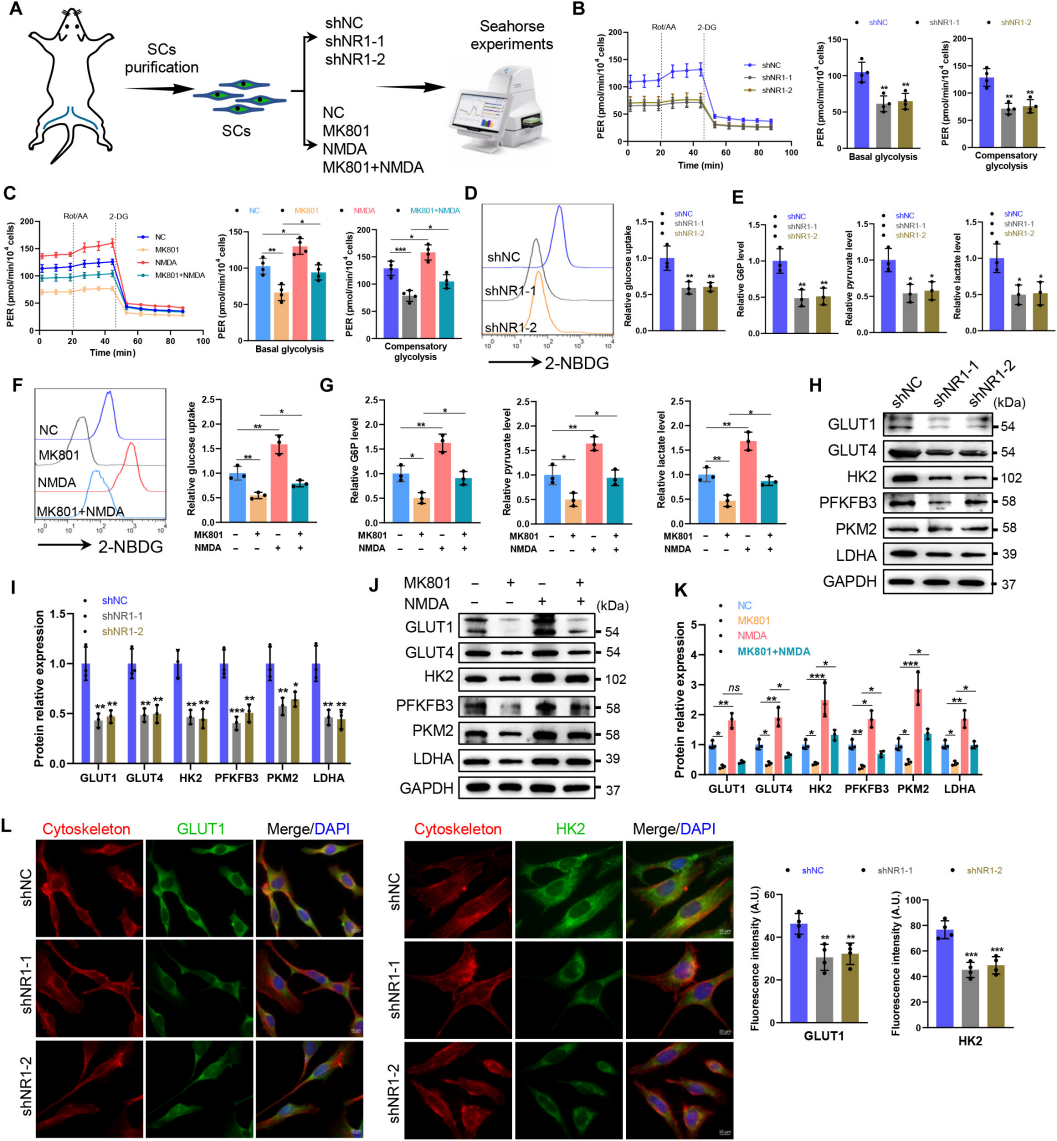
NMDA receptor regulates the glycolytic flux in SCs. (A) Schematic diagram of Seahorse experiments on purified SCs isolated from C57BL/6J mouse nerves. (B and C) The extracellular acidification rate (ECAR) of SCs was determined by a Seahorse XFe24. Glycolytic activity parameters, including basal glycolysis and compensatory glycolysis, were quantified. *n* = 4. (D and F) Glucose uptake was measured by flow cytometry using 2-NBDG. *n* = 3. (E and G) Glycolytic products in SCs, including glucose-6-phosphate (G6P), pyruvate, and lactate, were measured using chemiluminescence. *n* = 3. (H to K) Western blot analysis of the expression levels of glycolysis-related genes, including GLUT1, GLUT4, HK2, PFKFB3, PKM2, and LDHA, in SCs. *n* = 3. (L) Representative immunofluorescence and quantification of GLUT1 and HK2 in NR1-deficient SCs and control SCs. Scale bar, 10 μm. *N* = 4, *n* ≥ 10 fields/group. *ns*, not significant, *P < 0.05, **P < 0.01, ***P < 0.001.

Targeted metabolomics revealed impaired energy metabolism in NR1-deficient SCs, prompting further investigation using Seahorse experiments (Fig. 3A). Extracellular acidification rate (ECAR) measurements demonstrated that NMDA receptor inhibition (via NR1 knockdown or MK801) markedly reduced basal and compensatory glycolysis (Fig. 3B and C). Conversely, NMDA receptor activation enhanced glycolytic parameters and rescued MK801-induced deficits (Fig. 3C). Consistent with these findings, NR1 knockdown or MK801 treatment diminished glucose uptake and intracellular levels of glucose-6-phosphate, pyruvate, and lactate (Fig. 3D to G). NMDA receptor activation markedly increased these parameters and restored MK801-induced impairments (Fig. 3F and G).

Mechanistically, NMDA receptor inhibition down-regulated mRNA and protein levels of glucose transporter 1/4 (GLUT1/4) and glycolytic enzymes (hexokinase 2 [HK2], 6-phosphofructo-2-kinase/fructose-2,6-biphosphatase 3 [PFKFB3], pyruvate kinase M2 [PKM2], and lactate dehydrogenase A [LDHA]), whereas NMDA activation up-regulated their expression (Fig. 3H to L and Fig. S5). Notably, NMDA rescued MK801-induced suppression of GLUT4 expression and glucose uptake, restoring downstream glycolytic enzyme levels (Fig. 3J and K and Fig. S5B). These results establish NMDA receptors as key regulators of SC glycolytic flux through transcriptional and posttranslational control.

### NMDA receptors sustain oxidative metabolism via mitochondrial integrity

**Fig. 4.**
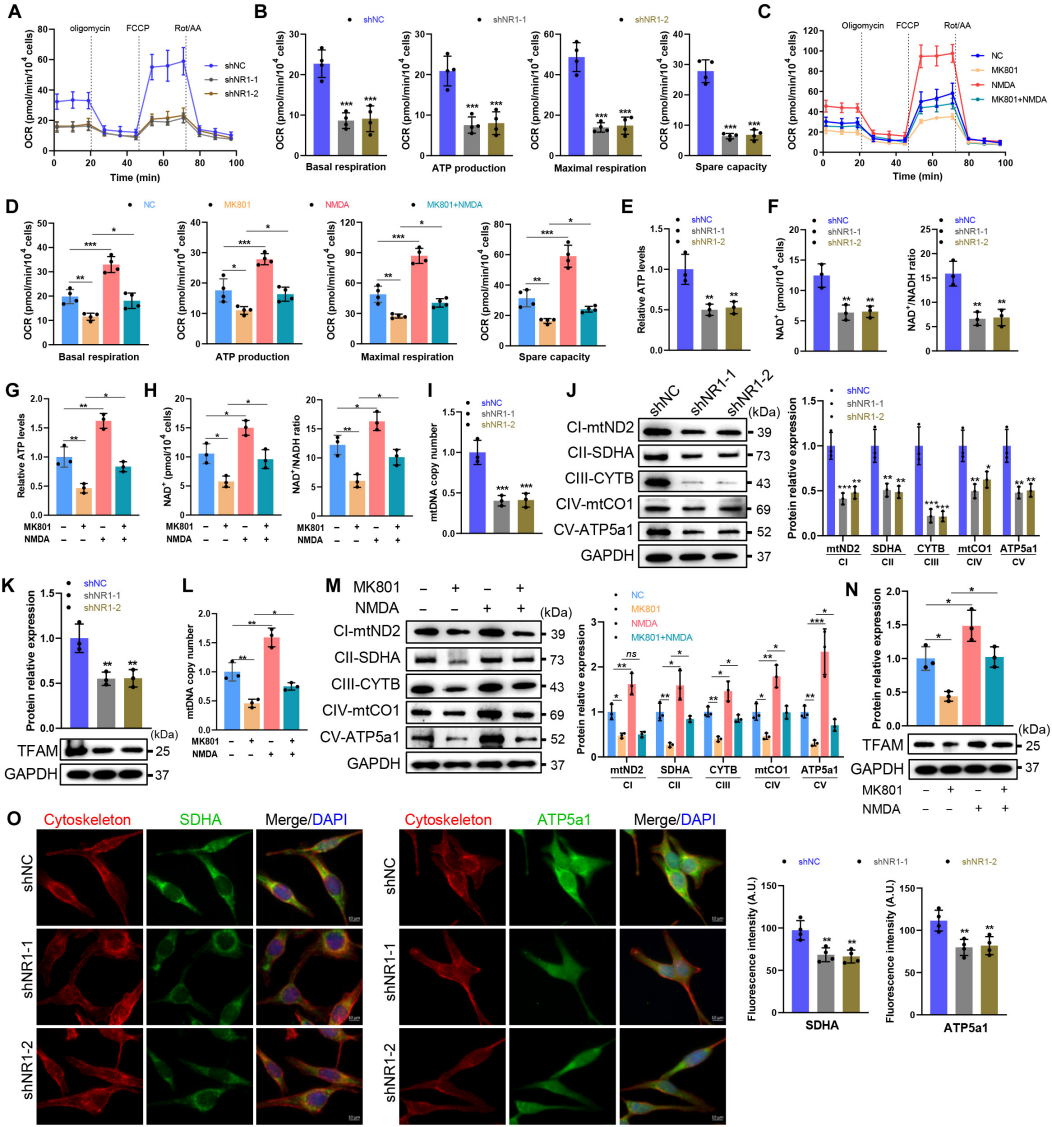
NMDA receptor regulates the oxidative metabolism in SCs. (A to D) The oxygen consumption rate (OCR) of SCs was determined by a Seahorse XFe24. Mitochondrial activity parameters, including basal respiration, ATP production, maximal respiration, and spare capacity, were quantified. *n* = 4. (E and G) Measurement of ATP production in SCs by chemiluminescence. *n* = 3. (F and H) The levels of NAD^+^ and the ratio of NAD^+^ to NADH in SCs were determined by chemiluminescence. *n* = 3. (I and L) qRT-PCR analysis of mtDNA copy number levels in SCs. *n* = 3. (J and M) Western blot analysis of the expression levels of mitochondrial electron transport chain (ETC) components, including mt-ND2, SDHA, mt-CYTB, mt-CO1, and mt-ATP5a1, in SCs. CI to CV represent the mitochondrial ETC complex I to V. *n* = 3. (K and N) Western blot analysis of the expression levels of TFAM in SCs. *n* = 3. (O) Representative immunofluorescence and quantification of SDHA and mt-ATP5a1 in NR1-deficient SCs and control SCs. Scale bar, 10 μm. *N* = 4, *n* ≥ 10 fields/group. *ns*, not significant, *P < 0.05, **P < 0.01, ***P < 0.001.

Given the interplay between glycolysis and oxidative phosphorylation, we assessed mitochondrial respiration [18,19]. Oxygen consumption rate (OCR) analysis revealed that NMDA receptor inhibition reduced basal respiration, ATP production, maximal respiration, and spare capacity, while NMDA activation increased these parameters and rescued MK801-induced deficits (Fig. 4A to D). The NAD^+^/NADH ratio is crucial for maintaining redox balance and ATP synthesis [20]. NR1 deficiency or MK801 treatment decreased NAD^+^/NADH ratios and intracellular ATP, which were restored by NMDA (Fig. 4E to H).

Mitochondrial respiration relies on electron transport chain (ETC) complexes encoded by nuclear and mitochondrial DNA [21]. NR1 deficiency or MK801 treatment reduced mitochondrial DNA (mtDNA) copy number and ETC components (e.g., mt-ND2, SDHA, mt-CYTB, mt-CO1, and mt-ATP5a1), while NMDA activation increased these parameters (Fig. 4I, J, L, M, and O and Fig. S6A, B, and E). NMDA also rescued MK801-induced suppression of SDHA, mt-CYTB, mt-CO1, and mt-Atp5a1 expression (Fig. 4M and Fig. S6B). Similarly, mitochondrial transcription factor A (TFAM), crucial for mtDNA replication, was down-regulated by NMDA receptor inhibition and restored by NMDA (Fig. 4K and N and Fig. S6C to E). These findings demonstrate that NMDA receptors preserve oxidative metabolism by maintaining mitochondrial genomic and structural integrity.

### NMDA receptor deficiency induces mitochondrial damage and dynamic imbalance

**Fig. 5.**
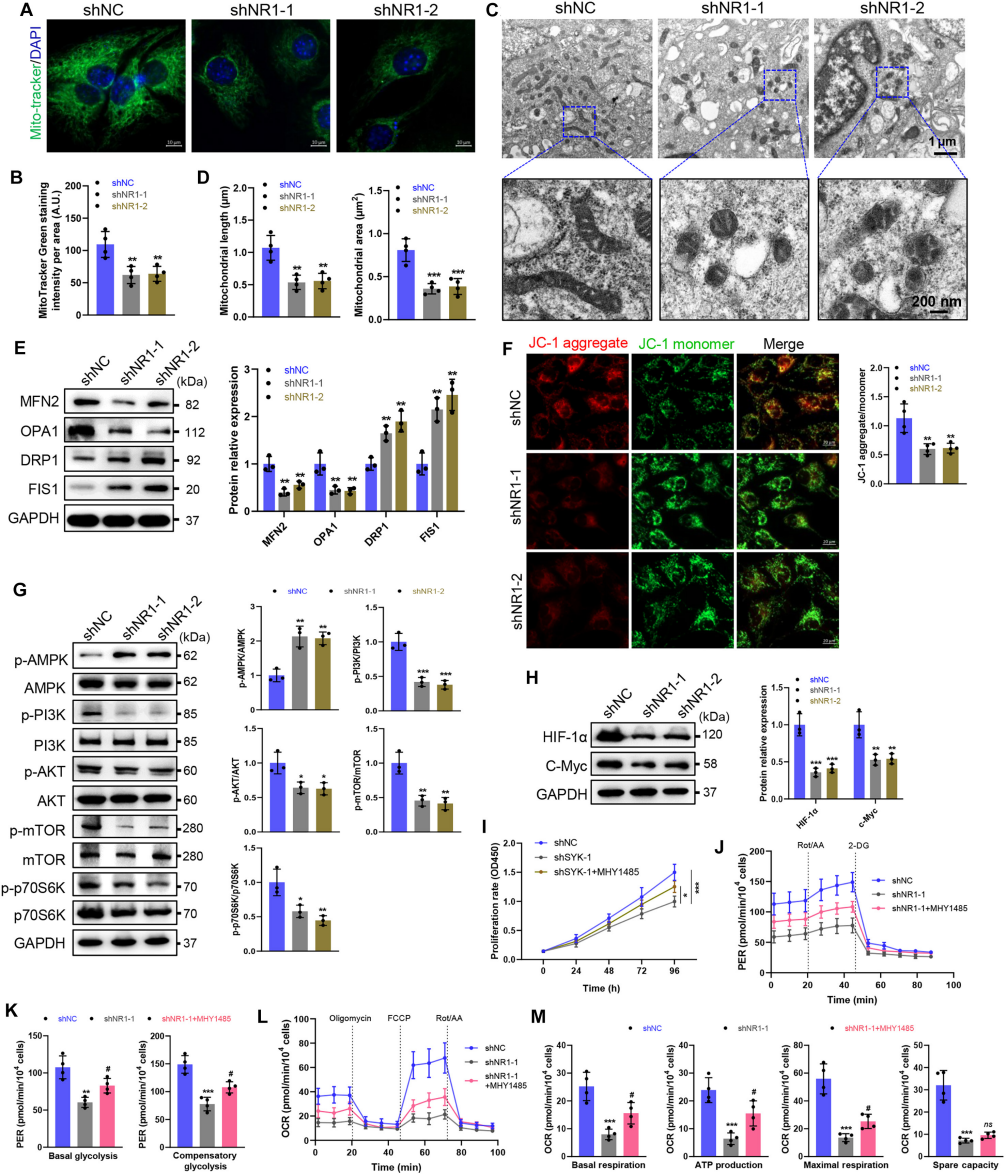
NMDA receptor deficiency induces mitochondrial damage in SCs. (A) Representative fluorescence images of mitochondria in SCs stained with MitoTracker Green. Scale bars, 10 μm. (B) The mitochondrial intensity was quantified, and the results are presented as the number of mitochondria. *N* = 4, *n* ≥ 10 fields/group. (C) Representative electron microscopy images of mitochondria in SCs. Upper scale bar, 1 μm; lower scale bar, 200 nm. (D) Quantification of mitochondrial length and area in SCs. *N* = 4, *n* ≥ 10 fields/group. (E) Western blot analysis of the levels of MFN2, OPA1, DRP1, and FIS1 in SCs. *n* = 3. (F) JC-1 probe staining and quantification of MMP in SCs. JC-1 aggregates indicate a high MMP while JC-1 monomers indicate a low MMP. Scale bars, 20 μm. *N* = 4, *n* ≥ 10 fields/group. (G) Western blot analysis of the levels of phosphorylated (Thr172) and total AMPK, phosphorylated (Tyr607) and total PI3K, phosphorylated (Ser473) and total AKT, phosphorylated (Ser2448) and total mTOR, and phosphorylated (Thr389/Thr412) and total p70S6K in SCs. *n* = 3. (H) Western blot analysis of the levels of HIF-1α and c-Myc in SCs. *n* = 3. (I to M) NR1-deficient SCs (shNR1-1) were treated with mTOR activator MHY1485 (5 μM). (I) Cell proliferation was determined by a CCK-8 assay. *n* = 4. (J to M) ECAR and OCR were measured by Seahorse experiments. Quantitative analysis of basal glycolysis and compensatory glycolysis in ECAR (K) and basal respiration, ATP production, maximal respiration, and spare capacity in OCR (M). *n* = 4. *P < 0.05, **P < 0.01, ***P < 0.001, compared with shNC; ^#^*P* < 0.05, compared with shNR1-1.

Metabolic disruptions in NR1-deficient SCs prompted assessment of mitochondrial integrity. MitoTracker staining showed reduced mitochondrial mass (Fig. 5A and B). Transmission electron microscopy (TEM) revealed fragmented mitochondria with cristae disorganization and membrane swelling (Fig. 5C and D). Analysis of mitochondrial dynamics proteins showed that NR1 deficiency reduced the fusion proteins mitofusin 2 (MFN2) and optic atrophy 1 (OPA1), while increasing the fission proteins dynamin-related protein 1 (DRP1) and its mitochondrial adaptor fission 1 (FIS1) (Fig. 5E). JC-1 staining showed a decrease in mitochondrial membrane potential (MMP) (Fig. 5F), corroborating exacerbated mitochondrial fragmentation, which is consistent with imbalanced dynamics. NMDA receptor activation rescued mitochondrial activity (Fig. S7A), suggesting a role in promoting fusion and suppressing excessive fission.

### NMDA receptor deficiency dysregulates AMPK and PI3K/AKT/mTOR signaling

The PI3K/AKT/mTOR pathway is a central regulator of cell growth, proliferation, and metabolism [19,22]. It plays a key role in promoting glycolysis and oxidative phosphorylation, critical for energy production in SCs during peripheral nerve regeneration [2,23]. Given the involvement of AMPK signaling in metabolic pathway alterations caused by NMDA receptor deficiency, along with the critical roles of AMPK and PI3K/AKT/mTOR pathways in energy metabolism, we investigated their interplay with NMDA receptors. NR1 deficiency increased AMPK phosphorylation while suppressing PI3K, AKT, and mTOR activation, leading to reduced p70S6K activity (Fig. 5G). Protein levels of metabolic regulators HIF-1α and c-Myc were markedly diminished in NR1-deficient SCs (Fig. 5H), indicating transcriptional dysregulation.

To confirm the role of PI3K/AKT/mTOR signaling, NR1-deficient SCs were treated with the mTOR activator MHY1485. The results showed that MHY1485 rescued cell proliferation (Fig. 5I) and up-regulated HIF-1α/c-Myc expression (Fig. S7B). ECAR analysis showed rescued basal and compensatory glycolysis (Fig. 5J and K), with qRT-PCR confirming increased glycolytic gene expression (Fig. S7C). MHY1485 also enhanced mitochondrial respiration, increasing mtDNA copy number, TFAM expression, and ETC components (Fig. S7D to F). OCR analysis confirmed elevated respiratory parameters (Fig. 5L and M). JC-1 staining further showed restored MMP (Fig. S7G). Furthermore, NR1-deficient SCs were treated with the HIF-1α activator deferoxamine to identify the protective role of HIF-1α/c-Myc signaling. The results showed that deferoxamine rescued the expression of glycolysis-related genes such as c-Myc, GLUT1, HK2, PKM2, LDHA, and cell growth-related genes such as vascular endothelial growth factor and Cyclin D1 (Fig. S8). Collectively, NMDA receptor deficiency disrupts energy metabolism by hyperactivating AMPK and suppressing PI3K/AKT/mTOR and HIF-1α/c-Myc signaling. Pharmacological mTOR activation reverses these deficits, restoring metabolic homeostasis.

### NMDA receptors modulate ATR–Atg13-mediated mitophagy under metabolic stress

**Fig. 6.**
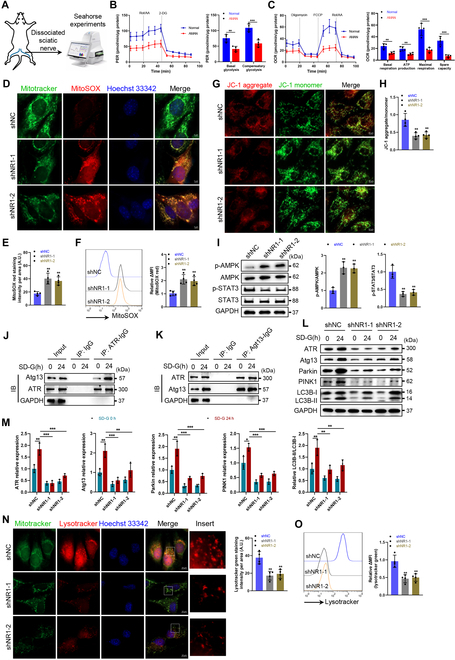
NMDA receptor deficiency impairs glucose starvation-induced mitophagy. (A) Schematic diagram of Seahorse experiments of dissociated sciatic nerves from normal and AMAN mice. (B and C) Quantitative analysis of basal glycolysis and compensatory glycolysis in ECAR (B) and basal respiration, ATP production, maximal respiration, and spare capacity in OCR (C). *n* = 4. (D to N) NR1-deficient SCs and control SCs were cultured in glucose starvation medium (SD-G). (D and E) Mitochondria were stained with MitoTracker Green and MitoSOX Red to evaluate the production of mitochondrial ROS. The MitoSOX Red fluorescence intensity was quantified. Scale bars, 5 μm. *N* = 4, *n* ≥ 10 fields/group. (F) Mitochondria were stained with MitoSOX Red and measured by flow cytometry. *n* = 4. (G and H) The MMP of SCs was measured by JC-1 probe, and the JC-1 aggregate/monomer ratio was quantified. Scale bars, 10 μm. *N* = 4, *n* ≥ 10 fields/group. (I) Western blot analysis of the levels of phosphorylated (Thr172) and total AMPK, and phosphorylated (Tyr705) and total STAT3 in SCs. *n* = 3. (J) Cells were cultured in SD-G for 0 and 24 h, and after which the lysates were immunoprecipitated with anti-ATR antibody and analyzed by Western blot using anti-Atg13 antibody. (K) Cells were cultured in SD-G for 0 and 24 h, and the lysates were immunoprecipitated with anti-Atg13 antibody and analyzed by Western blot using anti-ATR antibody. (L and M) Western blot analysis of the levels of ATR, Atg13, parkin, PINK1, and the C3II/LC3I ratio in SCs cultured in SD-G for 0 and 24 h. *n* = 3. (N) Mitochondria and lysosomes were stained with MitoTracker Green and LysoTracker Red. The LysoTracker Red fluorescence intensity was quantified. Scale bars, 10 μm. *N* = 4, *n* ≥ 10 fields/group. (O) Lysosomes were stained with LysoTracker Red and measured by flow cytometry. *n* = 4. **P < 0.01, ***P < 0.001.

To characterize metabolic alterations in peripheral nerve injury, we measured ECAR and OCR in sciatic nerves from the AMAN model (Fig. 6A). Injured nerves exhibited impaired glycolytic capacity, especially in basal glycolysis and compensatory glycolysis, along with diminished mitochondrial respiration, particularly in basal respiration, ATP production, maximal respiration, and spare capacity (Fig. 6A to C).

To mimic the energy-deprived microenvironment of injured nerves, SCs were subjected to glucose deprivation. NR1-deficient SCs displayed elevated mitochondrial reactive oxygen species (ROS) (Fig. 6D to F) and impaired MMP (Fig. 6G and H), indicating exacerbated mitochondrial dysfunction under metabolic stress.

Signal transduction and transcription activation factor 3 (STAT3) is a vital transcription factor of mitochondrial respiration, ROS production, and mitophagy [24]. Our investigation unveiled that NR1-deficient SCs exhibited increased AMPK phosphorylation and decreased STAT3 phosphorylation under glucose starvation (Fig. 6I), suggesting dysregulation of pathways critical for mitochondrial homeostasis. ATR and Atg13 are thought to facilitate autophagy induced by energy deprivation by regulating mitochondrial respiration [25,26]. Coimmunoprecipitation assays confirmed that ATR and Atg13 physically interact (Fig. 6J and K), forming a complex required for energy deprivation-induced autophagy. In glucose-starved SCs, mitochondrial damage triggers mitophagy, characterized by increased LC3-II/LC3-I ratio and up-regulation of autophagy-related proteins ATR, Atg13, parkin, and PTEN-induced putative kinase 1 (PINK1) (Fig. 6L and M). However, NR1 deficiency attenuated these responses, impairing autophagosome formation (Fig. 6L and M). Moreover, NMDA receptor deficiency reduced lysosomal abundance (Fig. 6N and O), further confirming the defective clearance of damaged mitochondria. Collectively, NR1 deficiency disrupts ATR–Atg13-mediated mitophagy during glucose starvation by hyperactivating AMPK and suppressing STAT3 signaling. This dysregulation exacerbates mitochondrial ROS accumulation and autophagic failure, highlighting NMDA receptors as pivotal regulators of SC adaptation to metabolic stress.

### NMDA receptors are essential for functional recovery in the AMAN mouse model

**Fig. 7.**
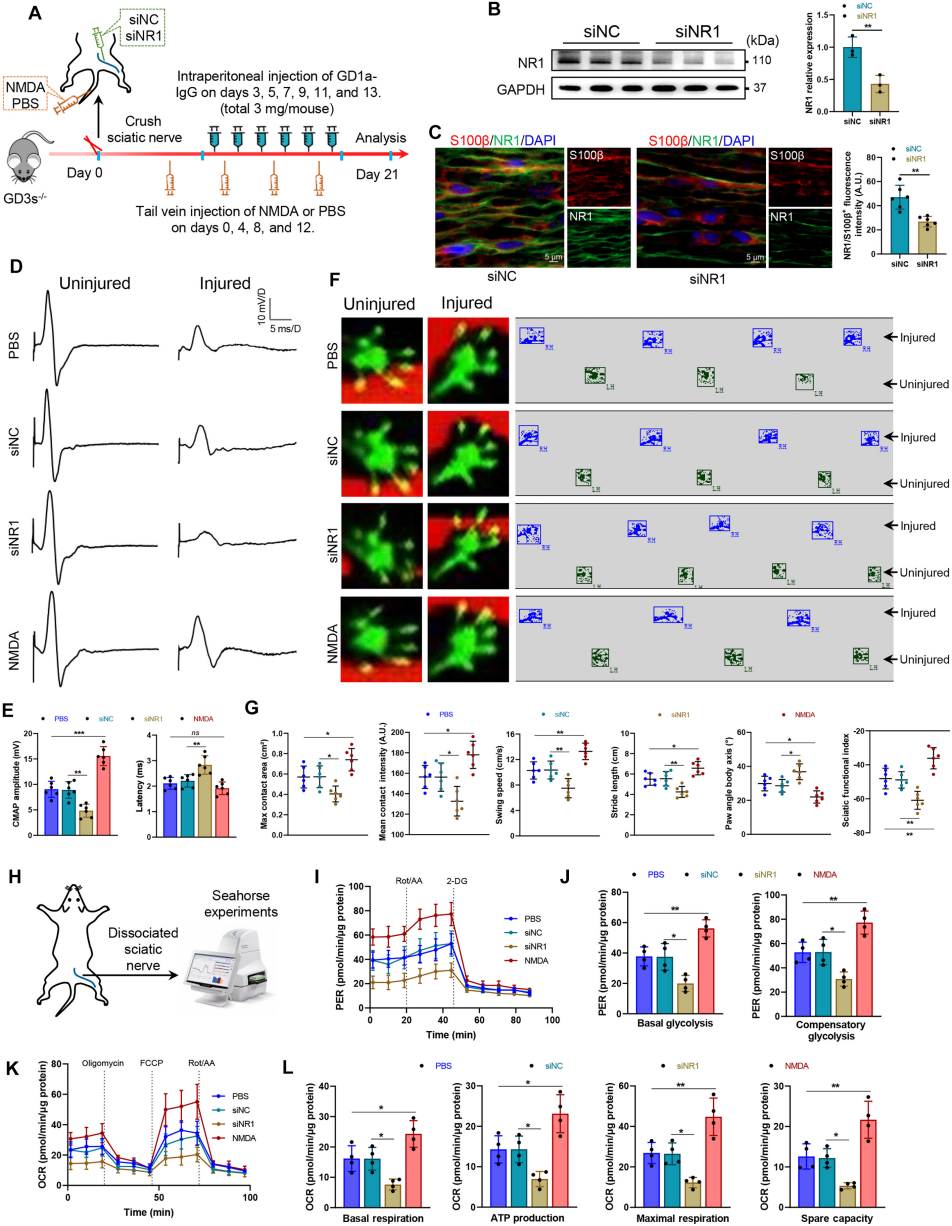
NMDA receptor regulates the recovery of PNS damage in an AMAN mouse model. (A) Schematic diagram of the experimental process in the mouse model of AMAN. Local injections of PBS, NMDA, siNC, or siNR1 were administered in the right sciatic nerve, followed by crushing. (B) Western blot analysis of the down-regulated NR1 expression induced by siNR1 in sciatic nerves. *n* = 3. (C) Representative immunofluorescence and quantification of NR1 in the sciatic nerves of AMAN mice injected with siNC or siNR1. S100β indicates SCs. The fluorescence intensity of NR1 in the S100β-positive region was statistically analyzed. Scale bars, 10 μm. *N* = 6, *n* ≥ 6 fields/group. (D and E) Electrophysiological tests were applied to evaluate nerve conduction in the sciatic nerve, and the CAMP amplitude and latency were quantified. *n* = 6. (F and G) Gait analysis was applied to evaluate motor function, and gait parameters and sciatic functional index were quantified. *n* = 6. (H) Schematic diagram of Seahorse experiments of dissociated sciatic nerves from AMAN mice injected with PBS, NMDA, siNC, or siNR1. (I to L) ECAR (I) and OCR (K) were measured by a Seahorse XFe24. Quantitative analysis of basal glycolysis and compensatory glycolysis in ECAR (J) and basal respiration, ATP production, maximal respiration, and spare capacity in OCR (L). *n* = 4. *ns*, not significant, *P < 0.05, **P < 0.01, ***P < 0.001.

To elucidate the role of NMDA receptors in the PNS, NR1-targeting small interfering RNA (siRNA) was locally administered to sciatic nerves followed by crush injury, or NMDA was administered via tail vein injection postcrush (Fig. 7A). Western blot confirmed efficient NR1 knockdown (Fig. 7B), and immunofluorescence staining colabeled with SC-specific markers S100β verified NR1 down-regulation in SCs (Fig. 7C). Electrophysiological assessments revealed that NR1 deficiency reduced compound muscle action potential (CMAP) amplitude and prolonged latency, whereas NMDA activation restored CMAP recovery (Fig. 7D and E). Gait analysis demonstrated worsened motor function in NR1-deficient mice, evidenced by increased paw angle deviation, reduced stride length, and impaired sciatic functional index (Fig. 7F and G). Conversely, NMDA activation facilitated the recovery of impaired motor function (Fig. 7F and G). Metabolic profiling via Seahorse assays linked these functional impairments to disrupted energy metabolism (Fig. 7H). NR1 deficiency impaired ECAR, especially in glycolysis and compensatory glycolysis, as well as OCR, particularly in basal respiration, ATP production, maximal respiration, and spare capacity (Fig. 7I to L). NMDA activation restored metabolic profiling (Fig. 7I to L). These findings establish that NMDA receptor signaling sustains energy metabolism in injured nerves, enabling functional recovery.

### NMDA receptors orchestrate metabolic and mitophagic adaptations during regeneration

**Fig. 8.**
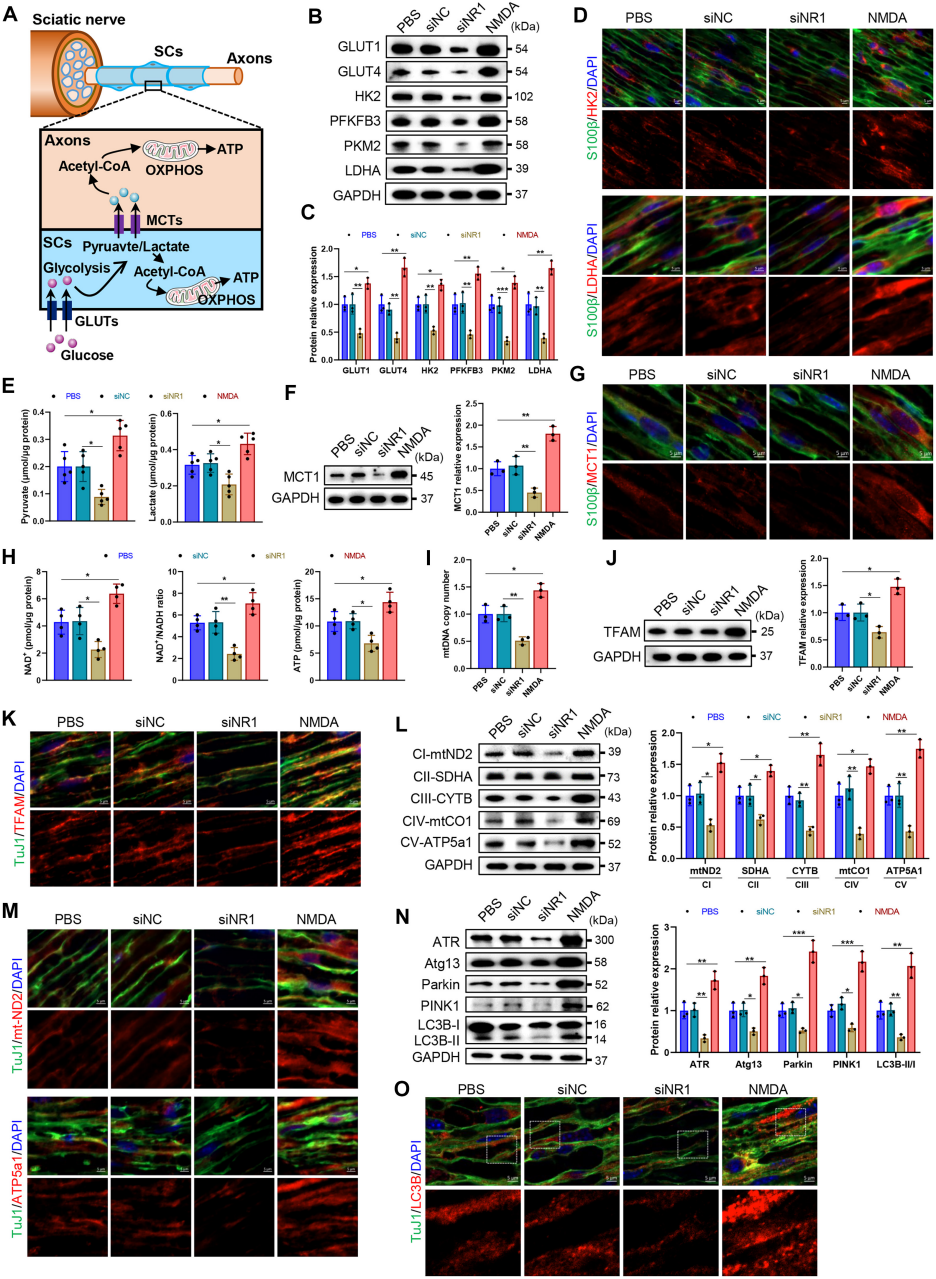
NMDA receptor regulates energy metabolism and autophagy in the sciatic nerve of AMAN mice. (A) Diagram illustrating the metabolism of SCs supporting axons. (B and C) Western blot analysis of the expression levels of GLUT1, GLUT4, HK2, PFKFB3, PKM2, and LDHA in the sciatic nerves of AMAN mice. *n* = 3. (D) Representative immunofluorescence of HK2 and LDHA in the sciatic nerves of AMAN mice. S100β indicates SCs. Scale bar, 5 μm. (E) Measurement of pyruvate and lactate in the homogenate supernatants of sciatic nerves from AMAN mice by chemiluminescence. *n* = 5. (F) Western blot analysis of MCT1 expression in the sciatic nerves of AMAN mice. *n* = 3. (G) Representative immunofluorescence of MCT1 in the sciatic nerves of AMAN mice. Scale bar, 5 μm. (H) The levels of NAD^+^ and the ratio of NAD^+^ to NADH, and ATP production in the homogenate supernatants of sciatic nerves from AMAN mice were measured by chemiluminescence. *n* = 4. (I) qRT-PCR analysis of mtDNA copy number in sciatic nerves from AMAN mice. *n* = 3. (J) Western blot analysis of TFAM expression in the sciatic nerves of AMAN mice. *n* = 3. (K) Representative immunofluorescence of TFAM in the sciatic nerves of AMAN mice. TuJ1 indicates neurons. Scale bar, 5 μm. (L) Western blot analysis of the expression levels of mt-ND2, SDHA, mt-CYTB, mt-CO1, and mt-Atp5a1 in the sciatic nerves of AMAN mice. *n* = 3. (M) Representative immunofluorescence of mt-ND2 and mt-Atp5a1 in the sciatic nerves of AMAN mice. Scale bar, 5 μm. (N) Western blot analysis of the levels of ATR, Atg13, parkin, PINK1, and the C3II/LC3I ratio in the sciatic nerves of AMAN mice. *n* = 3. (O) Representative immunofluorescence of LC3B in the sciatic nerves of AMAN mice, where diffuse cytoplasmic staining indicated LC3-I, and punctate staining indicated LC3-II. As shown, NMDA receptor deficiency impaired LC3B-II formation, indicating impaired autophagosome formation, while NMDA receptor activation promoted this process. Scale bar, 5 μm. *P < 0.05, **P < 0.01, ***P < 0.001.

SC-derived lactate, a glycolysis by-product, fuels axonal mitochondria via glia–axonal metabolic coupling (Fig. 8A) [2,3]. To investigate this mechanism, we first assessed the effect of NMDA receptors on glycolysis in injured sciatic nerves. NR1 deficiency markedly reduced the expression of GLUT1/4, HK2, PFKFB3, PKM2, and LDHA, whereas NMDA activation elevated these markers (Fig. 8B to D). Neurons can rapidly convert imported lactate into pyruvate for entry into the TCA cycle and oxidative phosphorylation [27]. Thus, pyruvate and lactate levels were measured and found to be reduced in the NR1-deficient group, while moderately elevated with NMDA activation (Fig. 8E). In line with this, monocarboxylate transporter 1 (MCT1) decreased in the NR1-deficient group and increased in the NMDA-activated group (Fig. 8F and G). Chemiluminescence measurements further corroborated these metabolic changes, demonstrating corresponding fluctuations in NAD^+^ levels, NAD^+^/NADH ratios, and ATP production in injured sciatic nerves (Fig. 8H).

Mitochondrial dynamics were similarly affected. NR1 deficiency markedly diminished mtDNA copy numbers, TFAM expression, and ETC components (mt-ND2, SDHA, mt-CYTB, mt-CO1, and mt-Atp5a1), while NMDA activation increased these levels (Fig. 8I to M). Mitophagy, critical for clearing damaged mitochondria, was impaired in NR1-deficient nerves, as shown by reduced ATR, Atg13, PINK1, parkin, and LC3-II/LC3-I ratios. NMDA activation enhanced these markers, indicating restored mitophagic flux (Fig. 8N and O). Collectively, these findings support the hypothesis that increased lactate/pyruvate transfer from SCs to neuronal axons boosts postinjury mitochondrial activity in the PNS, with NMDA receptors critically mediating this process.

### NMDA receptor activation as a therapeutic strategy to promote peripheral nerve regeneration

**Fig. 9.**
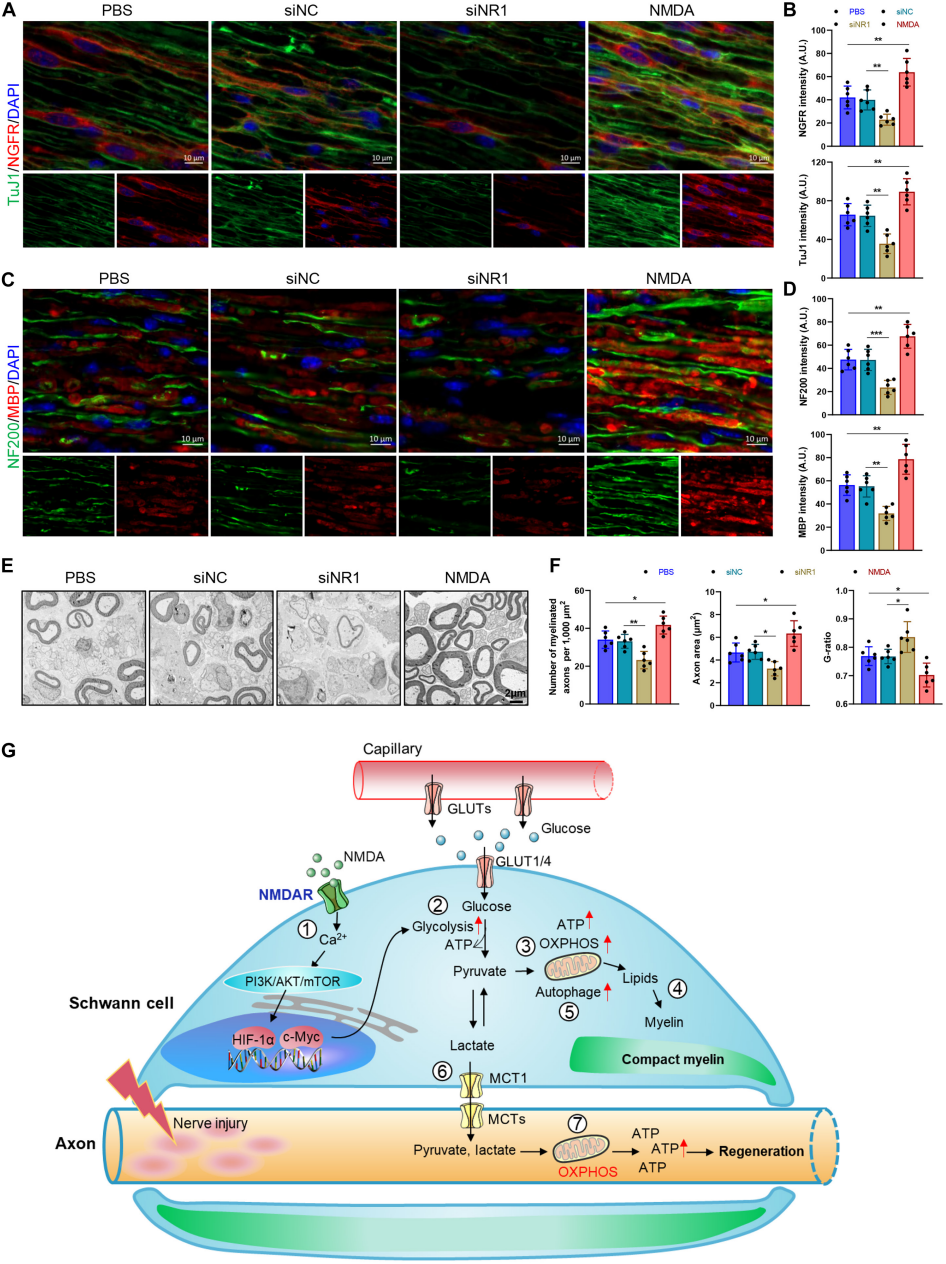
NMDA receptor supports regeneration after peripheral nerve injury. (A and B) Representative immunofluorescence and fluorescence intensity quantification of NGFR and TuJ1 in the sciatic nerves of AMAN mice. Scale bar, 10 μm. *N* = 6, *n* ≥ 6 fields/group. (C and D) Representative immunofluorescence and fluorescence intensity quantification of NF200 and MBP in the sciatic nerves of AMAN mice. Scale bar, 10 μm. *N* = 6, *n* ≥ 6 fields/group. (E) Representative TEM images of sciatic nerves of AMAN mice. (F) Quantification of the number of myelinated axons, axonal area and *g* ratio of nerve fibers. *G* ratio of the myelinated nerve fiber was calculated as the diameter of the axon divided by the diameter of the whole myelinated fiber. *P < 0.05, **P < 0.01, ***P < 0.001. (G) Schematic illustration of NMDA receptor regulating energy metabolism in SCs and supporting axonal regeneration. After injury, NMDA receptor (NMDAR) is activated in response to the high-energy metabolic demands of axons, activating PI3K/AKT/mTOR signaling and downstream HIF-1α/c-Myc (1), causing the entry of additional glucose transporters into SCs and up-regulation of glucose uptake (2). Glucose is converted into pyruvate in glycolysis (2). Glycolytic products are initially used for oxidative phosphorylation (OXPHOS) to produce ATP (3) and lipid synthesis (4). Mitophagy clears damaged mitochondria (5). SCs release lactate (or pyruvate) to fuel the axonal compartment (6) for mitochondrial ATP production to support nerve regeneration (7).

Given the pivotal role of NMDA receptors in modulating axonal mitochondrial activity through SC–axonal metabolic coupling, we explored the influence of NMDA receptor on PNS regeneration. NR1 deficiency reduced the expression of nerve growth factor receptor (NGFR), a marker for SCs with regenerative capacity, along with decreased neuronal production (Fig. 9A and B). The NR1-deficient group also exhibited diminished neurofilament 200 (NF200) and myelin basic protein (MBP) levels, indicating compromised axonal growth support and SC myelination (Fig. 9C and D). Conversely, NMDA activation was associated with increased expression of regenerative SC markers and increased axonal/myelin proteins, suggesting a beneficial role in postinjury PNS regeneration (Fig. 9A to D). TEM revealed fewer myelinated axons, smaller axonal cross-sectional areas, and elevated *g* ratios in NR1-deficient nerves, reflecting impaired axonal regeneration and myelination (Fig. 9E and F). NMDA activation reversed these deficits, increasing the count and area of myelinated axons, reducing the *g* ratio (Fig. 9E and F). These structural improvements correlated with restored electrophysiological and motor function, underscoring NMDA receptors as pivotal mediators of SC-axonal metabolic coupling and regeneration.

## Discussion

Neurons critically depend on glial metabolic support due to their limited intrinsic energy reserves [28–30]. SCs, the primary glia of the PNS, dynamically adapt their metabolism to sustain axonal survival and facilitate postinjury repair [2,16,31]. However, the molecular mechanisms driving SC metabolic plasticity during nerve regeneration remain poorly understood. In this study, we identified NMDA receptors as key regulators of SC metabolic reprogramming during nerve repair. Using a mouse model of AMAN, we observed a marked decrease in NMDA receptor expression in SCs of the sciatic nerve following neuropathy onset. This aligns with clinical observations from sural nerve biopsies of patients with immune-mediated peripheral neuropathies, suggesting a neuroprotective role of NMDA receptors that could be therapeutically exploited.

Our results indicate that NMDA receptor deficiency disrupts SC energy metabolism and mitophagy, leading to mitochondrial damage and impaired axonal survival. Conversely, pharmacological activation of NMDA receptors restores metabolic homeostasis and accelerates axonal regeneration. We found that NR1 down-regulation in SCs postinjury is a transient yet prolonged process, with a marked reduction in NR1 expression during the early phase of injury (24 to 72 h). This suggests that this period may represent a critical therapeutic window for NMDA receptor modulation. Restoring NR1 expression or enhancing NMDA receptor activity during this early phase could potentially promote peripheral nerve regeneration. Meanwhile, the partial recovery of NR1 expression at later stages implies that NMDA receptor modulation may still play a supportive role in the later phases of regeneration. The synchronous down-regulation of NR2C/D with NR1 postinjury suggests coordinated suppression of functional receptor complexes.

NMDA receptors regulate calcium influx, a process critical for synaptogenesis and signal transduction in both glial cells and neurons [32,33]. This calcium signaling cascade governs mitochondrial and nuclear functions essential for energy metabolism and cellular proliferation [34]. Our data demonstrate that NMDA receptor deficiency disrupts intracellular calcium homeostasis, whereas receptor activation restores this balance. Notably, impaired SC proliferation in NMDA receptor-deficient cells further corroborates the functional link between calcium signaling and metabolic regulation.

Energy metabolomics revealed that NMDA receptor deficiency markedly reduces glycolytic metabolites, TCA cycle intermediates, nucleotides, and amino acids in SCs, indicative of compromised glycolysis and oxidative phosphorylation. These observations were further validated by impaired ECAR and OCR, confirming a systemic impairment of energy metabolism. Notably, receptor activation rescued these metabolic deficits, highlighting NMDA receptors as gatekeepers of SC metabolic reprogramming.

Glial-mediated metabolic support critically depends on glucose uptake through GLUT transporters and subsequent lactate shuttling to axons [13,35,36]. We found that NMDA receptor deficiency down-regulates GLUT1/4 expression in SCs, impairing glucose uptake and glycolysis initiation. This metabolic dysfunction is further evidenced by reduced expression of glycolytic enzymes and diminished production of pyruvate and lactate. These metabolic disruptions cascade to mitochondrial metabolism, reducing ATP and NAD^+^ levels and compromising MMP. These effects correlate with reduced mtDNA copy number, decreased mitochondrial ETC complexes, and altered mitochondrial morphology in NMDA receptor-deficient SCs. Notably, NMDA receptor activation rescues these defects, enhancing glycolytic flux, oxidative phosphorylation, and mitochondrial bioenergetics.

Our study further implicates NMDA receptors in regulating mitochondrial dynamics in SCs. NMDA receptor deficiency shifts the balance toward fission-dominated dynamics, marked by decreased MFN2 and OPA1 expression and increased DRP1/FIS1 levels. This excessive fragmentation drives respiratory inefficiency and ROS overproduction, consistent with our observations of cristae disorganization and impaired oxidative phosphorylation. NMDA receptor activation modulates DRP1 activity to regulate fission during synaptic plasticity in neurons [37], and a similar mechanism may operate in SCs. Mitochondrial fission is often a prerequisite for mitophagy, as damaged organelles are segregated for degradation [38,39]. However, chronic fission due to NMDA receptor deficiency likely overwhelms mitophagy clearance, exacerbating ROS accumulation. Thus, NMDA receptors orchestrate mitochondrial homeostasis by coupling fission–fusion equilibrium with mitophagy.

The PI3K/AKT/mTOR pathway, a central regulator of cellular metabolism and proliferation [22,23], intersects with NMDA receptor signaling in SCs. Metabolic “master regulators” such as HIF-1α and c-Myc tightly control glycolytic and oxidative phosphorylation gene networks [22,40]. We identify the PI3K/AKT/mTOR signaling pathway as a key regulator of NMDA receptor-mediated energy metabolism in SCs. NMDA receptor deficiency disrupts PI3K/AKT/mTOR signaling, destabilizing HIF-1α and c-Myc activity and impairing both glycolysis and oxidative phosphorylation. These deficits hinder protein synthesis and SC proliferation [2,41], consistent with metabolomic data showing compensatory AMPK pathway activation under energy stress. Importantly, mTOR activation restores impaired glycolytic flux, oxidative metabolism, and mitochondrial activity, and HIF-1α activation can rescue glycolytic genes and cell growth genes, consolidating the PI3K/AKT/mTOR axis and HIF-1α/c-Myc signaling as key effectors of NMDA receptor-mediated energy metabolism in SCs.

Sciatic nerve crush and GD1a-IgG-mediated neuropathological injury profoundly impair the energy metabolism of the sciatic nerves, necessitating metabolic adaptation for repair. Autophagy, critical for maintaining energy balance, is central to this adaptation [42,43]. Our findings suggest that NMDA receptors coordinate autophagy control through the ATR–Atg13–PINK1–parkin axis in SCs, a mechanism for mitochondrial quality control under energy deprivation. During starvation, ATR, a DNA damage sensor, initiates autophagy by facilitating Atg13 binding to the Atg1/17 kinase complex, which drives autophagosome formation [25,26,44]. Damaged mitochondria recruit PINK1 to their outer membrane, triggering parkin-mediated ubiquitination of mitochondrial substrates and subsequent mitophagy [45–47]. Lipidated LC3-II, a marker of autophagosome maturation [48,49], is reduced in NMDA receptor-deficient SCs, reflecting impaired mitophagy. Mechanistically, NMDA receptor deficiency exacerbates mitochondrial dysfunction by activating AMPK and impairing STAT3 signaling under metabolic stress. This impairment leads to elevated mitochondrial ROS and defective mitophagy. These defects may result in the accumulation of damaged mitochondria, ultimately impairing energy metabolism. Our data thus establish NMDA receptors as essential regulators of SC mitochondrial homeostasis during energy stress, with the NMDA receptor–ATR–Atg13 axis representing a novel pathway for mitochondrial quality control in peripheral nerve repair.

Our study provides compelling evidence that NMDA receptor deficiency markedly delays nerve conduction and motor function recovery in an AMAN mouse model. This delay correlates with disrupted glycolysis and oxidative metabolism in injured sciatic nerves. Conversely, NMDA receptor activation accelerates repair by enhancing SC metabolic plasticity. Recent research underlines the rapid activation of glycolysis in SCs immediately following acute nerve injury, particularly within the first 72 h, coinciding with the initiation of Wallerian degeneration [2]. While glycolytic activation is critical during this early phase, our data demonstrate that sustained regeneration requires a metabolic shift to oxidative phosphorylation in reparative SCs. Lactate, a glycolytic by-product, serves dual roles: it fuels myelin lipid synthesis in SCs and acts as a neuronal energy substrate via MCTs. This glia–axonal metabolic coupling is indispensable, as evidenced by severe axonal degeneration following oligodendrocyte-specific MCT1 depletion or myelin channel disruption [5,35]. Our work extends this paradigm to peripheral nerves, showing that SC-derived lactate and pyruvate sustain axonal oxidative phosphorylation, directly linking SC metabolism to axonal regeneration.

The transient up-regulation of macrophage NMDA receptors in the early stages of injury may be related to the regulation of inflammatory response and immune cell infiltration, while sustained SC NMDA receptor suppression disrupts metabolic adaptation. Critically, NMDA receptors are required to orchestrate SC metabolic support to neurons by coordinating glycolysis, oxidative phosphorylation, and mitophagy. Glycolysis generates lipid precursors for myelin synthesis, while oxidative phosphorylation provides ATP for SC proliferation and remyelination. Pharmacological NMDA receptor activation in injured mice restored these processes, enhancing SC metabolic output and creating a regenerative niche for axons. Furthermore, NMDA receptor-driven mitophagy ensures efficient mitochondrial turnover in SCs, preventing ROS accumulation and maintaining metabolite production for axonal energy demands. These findings position NMDA receptors as central regulators of SC metabolic plasticity, bridging acute stress adaptation to long-term nerve repair.

While our findings establish NR1-containing NMDA receptors as key regulators of SC metabolism, the functional diversity of NMDA receptors in SCs remains unresolved. In our study, we primarily focused on NR1, which is the core subunit essential for forming functional NMDA receptors. However, the role of NR2 and NR3 subunits in modulating channel kinetics, calcium permeability, and downstream signaling should not be overlooked. In neurons, subunit composition dictates receptor properties: NR2A-containing receptors activate pro-survival pathways such as PI3K/AKT, whereas NR2B-containing receptors preferentially drive synaptic plasticity or stress responses [50,51]. Whether these subunit-specific roles are conserved in SCs remains unclear. Further investigation into the subunit composition of SC-specific NMDA receptors and their selective contributions to metabolic adaptation could reveal distinct therapeutic targets for enhancing SC-mediated repair.

Our clinical sample size is limited due to the challenges of obtaining nerve biopsies from patients with peripheral neuropathy. However, the observed reduction in NR1 expression on SCs in these patients is consistent with our findings from the animal model. Future studies with larger sample sizes and multi-center collaborations are needed to further validate these results.

While our study demonstrates that NMDA receptor activation in SCs enhances lactate/pyruvate production and MCT1-mediated transfer, coculture of primary neurons and SCs contributes to the direct validation of metabolites shuttling to axons. Nevertheless, our data provide compelling indirect evidence: NMDA receptor activation increases lactate/pyruvate production and MCT1 transporter expression in injured nerves; this increases correlate with restored mitochondrial respiration and ATP production in axons; and inhibition of this pathway exacerbated axonal degeneration and impaired functional recovery, as shown by electrophysiology and gait analysis. Future studies using neuron–glia cocultures and in vivo metabolic tracing will be essential to conclusively map the fate of SC-derived metabolites in axonal compartments.

The interventions employed in this study, including siRNA, shRNA, and tail vein injections, lack cell specificity, potentially affecting NMDA receptors in nontarget cells like neurons and macrophages. While our study focused on the role of NMDA receptors in SCs, the contributions of NMDA receptors in other cell types to peripheral nerve repair remain unclear. The siRNA and shRNA methods, though useful, risk off-target effects and partial knockdown, which may undermine the mechanistic conclusions about SC-autonomous effects. To overcome these limitations, future research could enhance target specificity by developing peripherally restricted NMDA receptor agonists or using localized delivery with cell-specific promoters. Conditional genetic models could also be employed for precise control of NMDA receptor expression in SCs. These models would allow for the temporal and spatial control of NMDA receptor expression, enabling more precise mechanistic studies. Additionally, exploring NMDA receptor functions in other cells using conditional knockouts and cell type-specific agonists/antagonists, combined with single-cell sequencing, would provide a more comprehensive understanding of their roles in peripheral nerve regeneration.

Motor axons in the PNS are primarily cholinergic, but several potential sources of glutamate could activate NMDA receptors on SCs. Motor neurons may release glutamate in addition to acetylcholine at the neuromuscular junction [52,53]. SCs themselves can synthesize and release glutamate, which may contribute to autocrine or paracrine signaling [52]. Infiltrating immune cells like macrophages after nerve injury can also release glutamate [54]. Further studies are needed to clarify the specific sources of glutamate that activate NMDA receptors on SCs during peripheral nerve injury and repair.

While our study emphasizes the critical role of NMDA receptors in SC metabolic reprogramming and mitophagy, other indicators may also undergo substantial changes following peripheral nerve injury. A broader exploration using multi-omics techniques, such as transcriptomics, proteomics, and metabolomics, could provide a more comprehensive understanding of the molecular mechanisms involved in peripheral nerve regeneration. Transcriptomics could reveal changes in gene expression, proteomics could identify key proteins involved in injury response, and metabolomics could elucidate metabolic alterations beyond those directly regulated by NMDA receptors. Integrating these multi-omics approaches would offer a systems-level view of the complex processes underlying peripheral nerve repair. Since our conclusions are based on murine models, which may not fully reflect human SC biology, future studies should validate these mechanisms in human-derived SCs or alternative preclinical models to assess their broader applicability.

Our findings show that NMDA receptor activation promotes peripheral nerve regeneration and could complement current treatments for peripheral neuropathies. Current therapies, such as intravenous immunoglobulin, corticosteroids, and neurotrophic factors, mainly modulate immune responses or support neuronal survival. In contrast, NMDA receptor activation enhances SC metabolic plasticity and axonal energy support, potentially synergizing with existing treatments.

However, the clinical translation of NMDA receptor agonists requires rigorous safety assessment. Systemic NMDA receptor overactivation risks excitotoxicity, seizures, and cognitive dysfunction, particularly in the central nervous system [8,55]. Encouragingly, our data demonstrate functional recovery at doses that did not induce overt behavioral abnormalities in mice, suggesting a manageable safety margin. Future studies should develop peripherally restricted agonists or localized delivery methods to mitigate off-target risks. Clinical trials will be necessary to evaluate the safety and efficacy of NMDA receptor modulation in humans, with careful monitoring for adverse effects and exploration of optimal dosing regimens to balance therapeutic benefits and risks. Combining NMDA receptor agonists with current therapies may allow for lower doses of each treatment, potentially reducing side effects while enhancing regenerative outcomes.

In conclusion, our findings establish NMDA receptors as central regulators of SC metabolic reprogramming and mitochondrial quality control during peripheral nerve repair. By coordinating glycolysis, oxidative phosphorylation, and mitophagy in SCs, NMDA receptors facilitate glia–axonal metabolic coupling to support axonal regeneration (Fig. 9G). These findings align with emerging paradigms of SC-axon metabolic interdependence in maintaining neuronal function. By enhancing SC metabolic plasticity and axonal energy support, targeted modulation of this pathway could advance treatment for peripheral neuropathies.

## Materials and Methods

### Cell culture and treatment

Primary SCs were isolated from sciatic nerves of postnatal day 3 to 5 mice using our established protocol [56]. Bilateral nerves were dissected under sterile conditions, stripped of epineurium, and finely minced. Tissue fragments were digested sequentially with 0.05% collagenase/dispase (Roche, Mannheim, Germany) for 1 h and 0.25% trypsin (Gibco, NY, USA) for 15 min at 37 °C in a 5% CO_2_ incubator. Dissociated cells were plated on poly-L-lysine/laminin-coated dishes and maintained in Dulbecco’s Modified Eagle's Medium/F12 medium supplemented with 10% fetal bovine serum, 1% penicillin/streptomycin (Gibco, NY, USA), 2 μM forskolin (Sigma, MA, USA), and 10 ng/ml heregulin-β-1 (PeproTech, NJ, USA). After 2 days of cultivation, half the medium was refreshed with 10 μM cytosine arabinoside (MCE, NJ, USA) for 2 days to suppress fibroblast proliferation, followed by a 3-day expansion in growth medium. Fibroblasts were selectively detached during passaging using 0.125% trypsin. SC purity was confirmed by anti-S100β and anti-NGFR antibody immunostaining.

Two lentiviral shRNA constructs (Obio Technology, Shanghai, China) targeting NR1 were utilized: shNR1-1: 5′-AAACCAGGCCAAUAAGCGACA-3′; shNR1-2: 5′-AATGTCCATCTACTCTGACAA-3′. A nontargeting shRNA was utilized as the negative control. Puromycin (2 μg/ml) was added 48 h posttransfection to select stably transduced cells.

SCs were treated with 100 μM NMDA (agonist) or 5 μM MK801 (antagonist) for 48 to 72 h. For rescue assays, cells were preincubated with MK801 (5 μM, 30 min) prior to NMDA exposure. Phosphate-buffered saline (PBS)-treated groups served as baseline controls.

### CCK-8 assays

SCs were plated in 96-well plates (3,000 cells/well) and subjected to designated treatments. At specified time points, Cell Counting Kit-8 (CCK-8) reagent (Dojindo, Kumamoto, Japan) was added to respective wells, followed by a 2- to 4-h incubation at 37 °C. Absorbance was quantified at 450 nm using a BioTek Cytation Hybrid microplate reader (Agilent, CA, USA).

### qRT-PCR

Total RNA was isolated from SCs or sciatic nerves using TRIzol reagent (Invitrogen, CA, USA) and reverse-transcribed into cDNA with a PrimeScript II 1st Strand cDNA Synthesis Kit (Takara, Beijing, China). qRT-PCR was performed on an ABI QuantStudio5 system (Thermo Fisher, Woodlands, Singapore) using UltraSYBR Mixture (CWBIO, Taizhou, China). Target gene expression was normalized to GAPDH and calculated via the 2^−ΔΔCt^ method. For mtDNA quantification, genomic DNA was extracted with a Genomic DNA Kit (Tiangen, Beijing, China). mtDNA copy number was determined by amplifying mitochondrial COX2 and normalizing to nuclear RPS18, as previously described [57]. Primer sequences are listed in Table S1.

### Immunofluorescence staining

Sciatic nerves were fixed in situ with 4% paraformaldehyde (PFA), dissected, and postfixed in 4% PFA for 2 h. Tissues were dehydrated in 30% sucrose for over 24 h and embedded in optimal cutting temperature compound (Sakura Finetek, Torrance, USA). Longitudinal sections (8 μm) were cut and permeabilized with 0.5% Triton X-100 for 20 min, blocked with 10% goat serum in PBS for 0.5 h, and incubated with primary antibodies overnight at 4 °C. After washing, sections were labeled with Alexa Fluor-conjugated secondary antibodies at room temperature for 1 h, mounted in antifade medium, and coverslipped. For cells, SCs were fixed with 4% PFA for 15 min, permeabilized, and blocked in 10% goat serum and 0.5% Triton X-100 in PBS for 0.5 h. Cells were incubated with primary antibodies overnight at 4 °C, followed by Alexa Fluor-conjugated secondary antibodies or iFluor 555-phalloidin (Yeasen Biotechnology, Shanghai, China) for 1 h. Nuclei were counterstained with Fluoroshield containing 4′,6-diamidino-2-phenylindole (DAPI; Abcam, MA, USA). Images were acquired using a confocal laser scanning microscope (LSM 800, Zeiss, Jena, Germany). Fluorescence intensity was quantified across ≥6 fields per sample using ImageJ (NIH, MD, USA). Antibody details are provided in Table S2.

### Western blotting

Protein lysates were isolated from SCs and sciatic nerve segments (crush site to distal trunk) using radioimmunoprecipitation assay buffer (Beyotime, Beijing, China). Lysate concentrations were quantified, resolved by sodium dodecyl sulfate-polyacrylamide gel electrophoresis, and electrophoretically transferred to polyvinylidene difluoride membranes. After blocking with 5% nonfat milk, membranes were incubated overnight at 4 °C with primary antibodies, followed by horseradish peroxidase-conjugated secondary antibodies. Protein bands were detected using ECL chemiluminescence reagent (Proteintech, Wuhan, China) and quantified by ImageJ. Antibody details are provided in Table S2.

### Targeted energy metabolomics

Energy metabolites were profiled via liquid chromatography-tandem mass spectrometry (MetWare Biotechnology, Wuhan, China). Cultured SCs were homogenized in 500 μl of ice-cold 80% aqueous methanol, vortexed, and flash-frozen in liquid nitrogen. After thawing on ice, samples were revortexed and centrifuged at 12,000 *g* for 10 min at 4 °C. Supernatants were incubated at −20 °C for 0.5 h, recentrifuged, and analyzed using an AB-SCIEX QTRAP 6500 mass spectrometer (AB-SCIEX, MA, USA). The chromatographic separation utilized an ACQUITY UPLC BEH Amide column with a mobile phase consisting of water with 10 mM ammonium acetate and 0.3% ammonium hydroxide (A) and 90% acetonitrile/water (B). The gradient elution program started at 95% B, decreased to 70% B at 8 min, further reduced to 50% B at 9 to 11 min, and returned to 95% B. The mass spectrometer operated in both positive and negative ion modes, with parameters optimized for scheduled multiple reaction monitoring to identify and quantify metabolites. A specific set of multiple reaction monitoring transitions was monitored for each period according to the metabolites eluted within this period. Data were acquired using Analyst software. Multiquant software was used to quantify all metabolites based on peak integration and retention time alignment. Metabolites were identified by normalizing to reference standards. The color bar’s scale of heatmap is normalized by unit variance scaling, also known as *Z*-score normalization. This method normalizes data by subtracting the mean of the original data and dividing by the standard deviation. The processed data conform to a standard normal distribution with a mean of 0 and a standard deviation of 1. Differential metabolites were defined by fold change (FC ≥2 or ≤0.5), variable importance in projection (VIP ≥1), and significance (*P* < 0.05). Enriched pathways were annotated using the KEGG database. Rich factor is the ratio of the number of differential metabolites in the corresponding pathway to the total number of metabolites annotated by the pathway.

### Seahorse experiment

ECAR and OCR were quantified using a Seahorse XFe24 analyzer (Agilent Technologies, CA, USA). For cells, treated SCs were seeded into poly-L-lysine-coated Seahorse XFe24 plates (25,000 cells/well) and equilibrated overnight. For tissues, sciatic nerves were dissected, epineurium-removed, and minced. Tissue fragments were enzymatically dissociated with StemPro Accutase (Gibco, NY, USA) supplemented with 0.1% DNase I (Roche, Mannheim, Germany). Dissociated cells were plated on poly-L-lysine-coated Seahorse plates, centrifuged at 200 *g* for 2 min with zero speed braking, and maintained in assay-specific media. ECAR and OCR measurements were performed using our established protocol [23]. Data were normalized to total cell count or protein content and analyzed using Wave Desktop 2.6 (Agilent Technologies).

### Ca^2+^ measurement

Cellular Ca^2+^ levels were assessed using Fluo-4 AM (Beyotime, Beijing, China). SCs were loaded with 2 μM Fluo-4 AM in serum-free medium in the dark for 0.5 h at 37 °C. After dye loading and washing, cells were exposed to 100 μM NMDA for 10 min or 5 μM MK801 for 0.5 h. For rescue experiments, MK801-pretreated cells were subsequently challenged with 100 μM NMDA for 10 min. Fluorescence intensity was quantified immediately using flow cytometry (Beckman, DxFLEX, CA, USA).

### Measurement of glycolytic metabolites

Glucose uptake was assessed using 2-[*N*-(7-nitrobenz-2-oxa-1,3-diazol-4-yl)amino]-2-deoxy-D-glucose (2-NBDG) (Abcam, MA, USA). SCs were washed with PBS, equilibrated in glucose-free medium, and incubated with 100 μM 2-NBDG in the dark for 0.5 h. Fluorescence intensity was quantified via flow cytometry.

Glucose-6-phosphate, lactate, and pyruvate levels were measured using commercial kits (Abcam, MA, USA) per manufacturer guidelines. Absorbance was recorded at 450 nm for glucose-6-phosphate and at 570 nm for lactate and pyruvate.

### Measurement of ATP and NAD^+^/NADH

Intracellular ATP was quantified with a luminescent ATP detection kit (Beyotime Biotechnology, Beijing, China). Cells were lysed in detergent-based buffer for 5 min, mixed with substrate solution, and agitated at 600 to 700 rpm for 5 min. Luminescence at 570 nm was measured after 10 min of dark incubation.

NAD^+^ and NADH levels were determined using a WST-8-based kit (Beyotime Biotechnology, Beijing, China). The measurements were performed using our established protocol [57,58].

### MMP measurement

MMP was determined using the JC-1 probe (Abcam, MA, USA). SCs were incubated with JC-1 working solution in the dark for 15 min at 37 °C, washed, and resuspended in fresh medium. Imaging was captured on Cell Discoverer 7 microscope (Zeiss, Jena, Germany). JC-1 aggregates (590 nm) and monomers (529 nm) were quantified at least 10 visual fields in each independent experiment using ImageJ. MMP was expressed as the aggregate-to-monomer fluorescence ratio.

### Mitochondrial ROS detection

Mitochondrial ROS levels were detected using MitoSOX Red (MCE, CA, USA). For immunofluorescence staining, SCs were costained with 2 μM MitoSOX Red and 100 nM MitoTracker Green (Thermo Fisher, MA, USA) in the dark for 0.5 h at 37 °C. After washing, cells were imaged by a Cell Discoverer 7 microscope. For flow cytometry, SCs were stained with 2 μM MitoSOX Red in the dark for 0.5 h at 37 °C. After washing, cells were analyzed via flow cytometry.

### Lysosome detection

Lysosomes were evaluated using LysoTracker Red (Beyotime Biotechnology, Beijing, China). For immunofluorescence staining, SCs were costained with 1 μM LysoTracker Red and 100 nM MitoTracker Green in the dark for 0.5 h at 37 °C. After washing, cells were imaged by a Cell Discoverer 7 microscope. For flow cytometry, SCs were stained with 2 μM LysoTracker Red in the dark for 0.5 h at 37 °C. After washing, cells were analyzed via flow cytometry.

### Transmission electron microscopy

SCs and postcrush distal sciatic nerve segments were fixed overnight in phosphate buffer containing 2% PFA and 2.5% glutaraldehyde, followed by postfixation in 1% osmium tetroxide for 1 h. Tissues were dehydrated through a graded ethanol series, embedded in epoxy resin (SPI, NY, USA), and sectioned (70 nm) using an RMC-PT-PC ultramicrotome (Tucson, AZ, USA). Ultrathin sections were stained with 2% uranyl acetate and 1% lead citrate, then imaged on a Tolas L 120C electron microscope (Thermo Fisher, Brno, Czech Republic). Mitochondrial length, cross-sectional area in SCs, axonal diameter, myelin thickness, and axonal cross-sectional area of myelinated fibers were quantified using ImageJ. Data from ≥6 fields per sample were included for statistical analysis.

### GD1a-IgG purification

Anti-GD1a IgG was purified from plasma of a GD1a-IgG-seropositive GBS patient. Plasma was prefiltered to remove particulates and dialyzed against phosphate buffer (pH 7.2). IgG was isolated using a HiTrap Protein G HP column (GE Healthcare, IL, USA) on an ÄKTA Pure system (Cytiva, Washington, USA). Antibodies were eluted with 0.2 M glycine (pH 2.8), neutralized with 1.0 M Tris-HCl (pH 9.0), buffer-exchanged into PBS, and concentrated to 10 mg/ml. All protocols were approved by the Ethics Committee of the Medical Science Research of Affiliated Hospital of Jining Medical University (No. 2022-08-B001).

### AMAN animal model and treatment

GD3s^−/−^ mice overexpressing ganglioside GD1a were generated on the C57BL/6J background (Cyagen Biosciences, Guangzhou, China). Animals were maintained in a specific pathogen-free facility under a 12-h light/dark cycle with ad libitum access to food and water. The AMAN model was established as previously described [59], with minor modifications. Briefly, on day 0, the right sciatic nerve of 12-week-old male mice was crushed for 30 s using carbon-coated fine forceps. Intraperitoneal injections of GM1-IgG (3 mg total dose per mouse) were administered on postoperative days 3, 5, 7, 9, 11, and 13. Behavioral, electrophysiological assessments and nerve harvesting were conducted at 21 days postsurgery.

To silence NMDA receptor expression, intraneural siRNA injections were performed using Entranster-in vivo reagent (Engreen, Beijing, China). The siRNA sequence targeting NR1 (siNR1) was 5′-AAACCAGGCCAAUAAGCGACAtt-3′, with a scrambled siRNA (siNC) serving as a negative control. For each mouse, a mixture of 2 μg of siRNA and 1 μl of Entranster-in vivo was incubated at room temperature for 15 min and injected intraneurally into the right sciatic nerve immediately prior to crush injury on the day of surgery. To activate NMDA receptors, mice received intravenous tail vein injections of 100 μl of 2 mmol/l NMDA in PBS on postoperative days 0, 4, 8, and 12. Control groups were injected with equivalent volumes of PBS. All procedures were approved by the Ethics Committee of the Medical Science Research of Affiliated Hospital of Jining Medical University (No. 2022B005).

### Electrophysiology

Mice were anesthetized using 2% to 3% isoflurane for sciatic nerve electrophysiological testing. The sciatic nerves were exposed, and stimulating electrodes were positioned meticulously at the nerve trunk. Recording electrodes were placed on the gastrocnemius muscle belly, while the reference electrode was positioned at the Achilles tendon and the ground electrode in the tail. To measure CMAP amplitude and latency, the nerve was stimulated at least 3 times with 0.1-ms pulses at 1 Hz.

### Catwalk gait

Motor function was assessed using an automated gait analysis system called CatWalk (Zhongshi Dichuang Technology, Beijing, China). High-speed cameras recorded footstep patterns as mice traversed a runway. Mice underwent adaptive training to acclimate to the setup. Parameters extracted from the data included stance phase metrics (max contact area, max intensity, and mean intensity), swing phase metrics (swing duration and swing speed), stride length, step cycle, paw angle relative to the body axis, body speed, and paw drag. The Sciatic Nerve Function Index (SFI) was calculated as a noninvasive indicator of recovery using the formula [60]: SFI = 118.9(ETS − NTS)/NTS − 51.2(EPL − NPL)/NPL − 7.5, where ETS and EPL denote experimental toe spread (distance between the first and fifth toes) and experimental print length (distance from the third toe to the heel), respectively; NTS and NPL represent corresponding normal-side values. Lower SFI values indicate greater sciatic nerve impairment.

### Statistical analysis

All statistical analyses were performed using GraphPad Prism 9. Data are represented as the mean ± standard deviation. Normality was assessed via the Shapiro–Wilk test. For comparisons between 2 groups, 2-tailed Student’s *t* tests were used. One-way analysis of variance with Bonferroni post-hoc correction was applied for comparisons involving 3 or more groups. A threshold of *P* < 0.05 was used to determine statistical significance.

## Data Availability

All data are available from the corresponding authors upon request.
