# Supplementary material for: NMDA Receptors Coordinate Metabolic Reprogramming and Mitophagy in Schwann Cells to Promote Peripheral Nerve Regeneration
Source: Research (Wash D C). 2025 Aug 5;8:0825. doi: 10.34133/research.0825 (PMC12322490; doi:10.34133/research.0825)
Supplement: Supplementary 1 — Figs. S1 to S8 Tables S1 and S2 [file research.0825.f1.zip › Supplemental figure.docx]

**Supplemental figure**

**
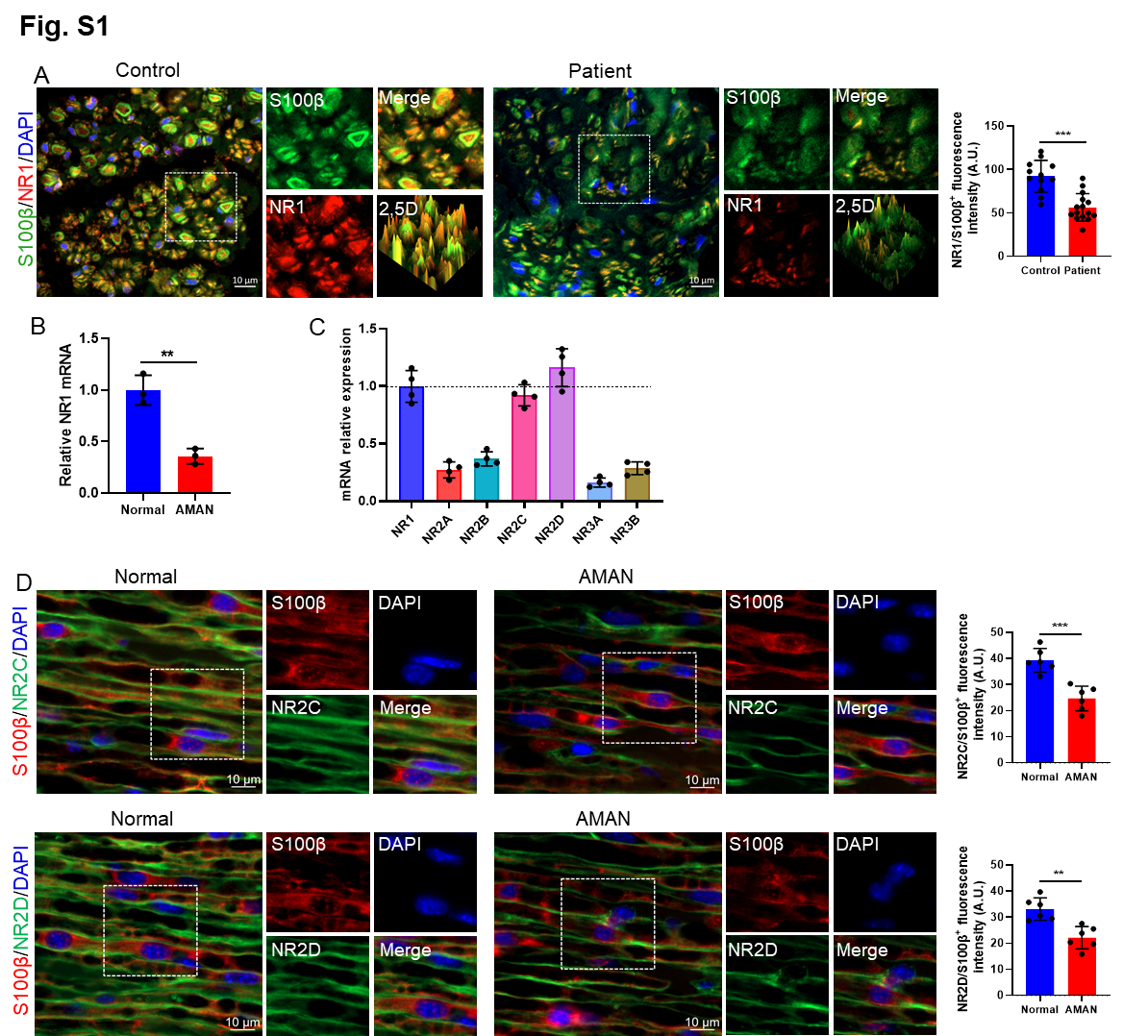
**

**Figure S1. Peripheral neuropathy reduces NMDA receptor expression on SCs**

(A). Representative immunofluorescence of NR1 in the sural nerve of biopsies from patients with immune peripheral neuropathy (n=15) and surgical injury amputation (n=12). S100β indicates SCs. The fluorescence intensity of NR1 in the S100β-positive region was statistically analyzed. Scale bar, 10 μm. (B). qRT-PCR analysis of relative mRNA expression of NR1 in the sciatic nerves of normal and AMAN mice. n = 3. (C). qRT-PCR analysis of relative mRNA expression of NMDA receptor subunits NR1, NR2 (A-D) and NR3 (A-B) in SCs. n = 4. (D). Representative immunofluorescence of NR2C and NR2D in the sciatic nerves of normal and AMAN mice. S100β indicates SCs. The fluorescence intensity of NR1 in the S100β-positive region was statistically analyzed. Scale bar, 10 μm. N = 6, n ≥ 6 fields/group. **p* < 0.05, ***p* < 0.01, ****p* < 0.001.

**
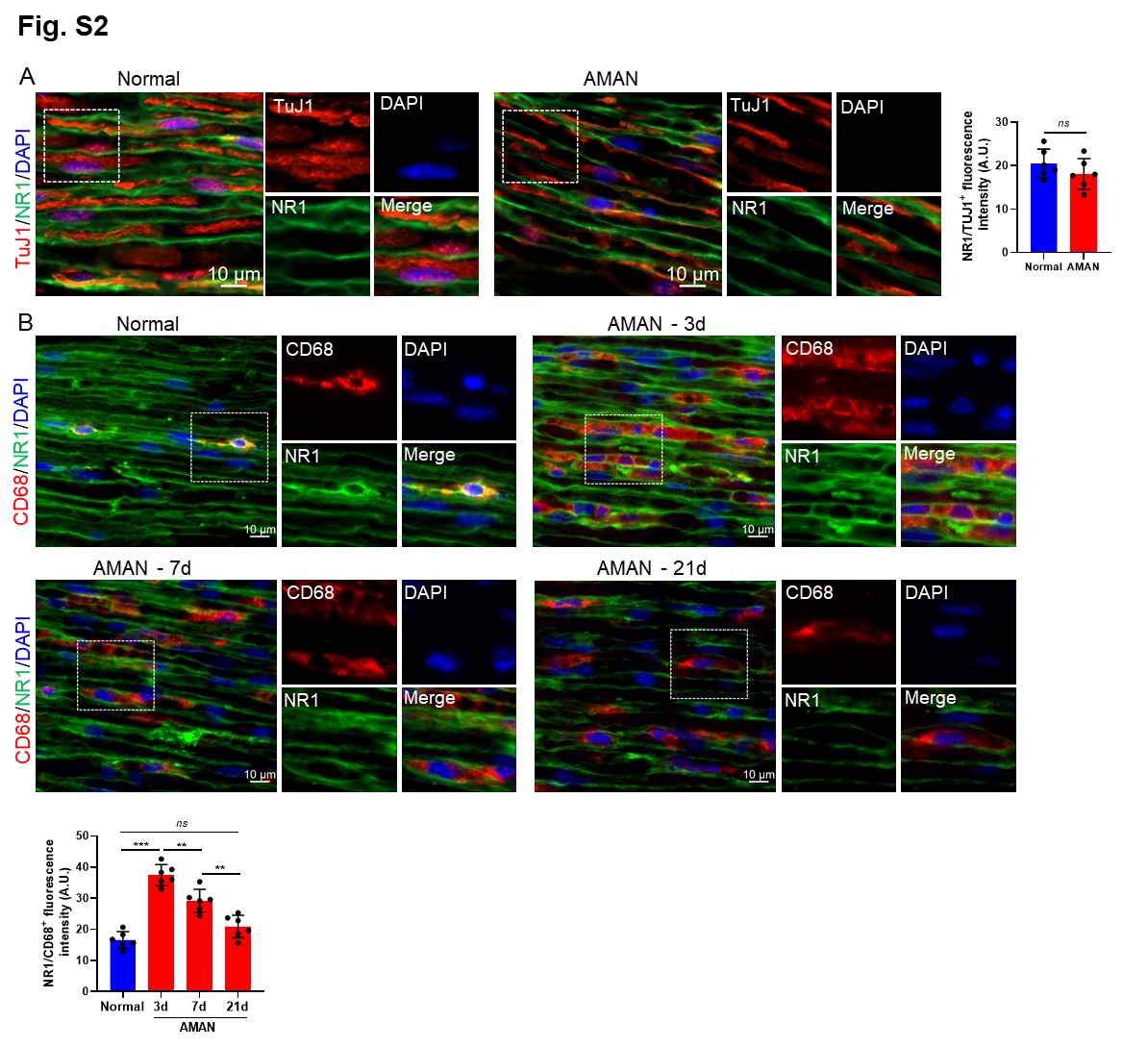
**

**Figure S2.** **Expression changes of NMDA receptors in neurons and macrophages in the lesion area of AMAN mice**

(A). Representative immunofluorescence of NR1 in the sciatic nerves of normal and AMAN mice. TuJ1 indicates neurons. The fluorescence intensity of NR1 in the TuJ1-positive region was statistically analyzed. Scale bar, 10 μm. N = 6, n ≥ 6 fields/group. (B). Representative immunofluorescence of NR1 in sciatic nerves of normal mice and AMAN mice on days 3, 7 and 21 after injury. CD68 represents macrophages. CD68 denotes macrophages. The fluorescence intensity of NR1 in the CD68-positive region was statistically analyzed. Scale bar, 10 μm. N = 6, n ≥ 6 fields/group. *ns*, not significant, ***p* < 0.01, ****p* < 0.001.

**
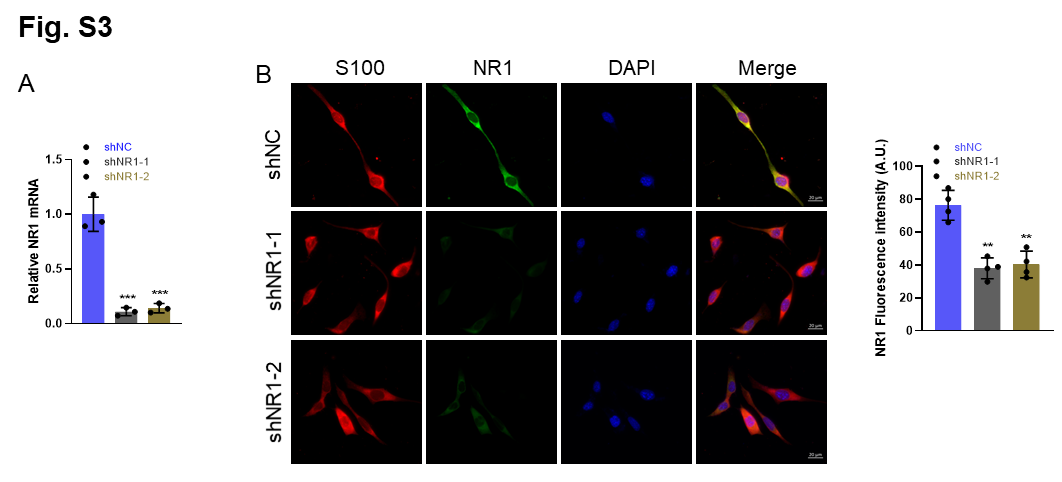
**

**Figure S3. Knockdown of NR1 in SCs**

(A). The efficiency of NR1 knockdown in primary SCs using NR1-specific shRNAs was identified by qRT-PCR. n = 3. (B). Representative immunofluorescence and quantification of NR1 in SCs. Scale bar, 20 μm. N = 4, n ≥ 10 fields/group. **p* < 0.05, ***p* < 0.01.


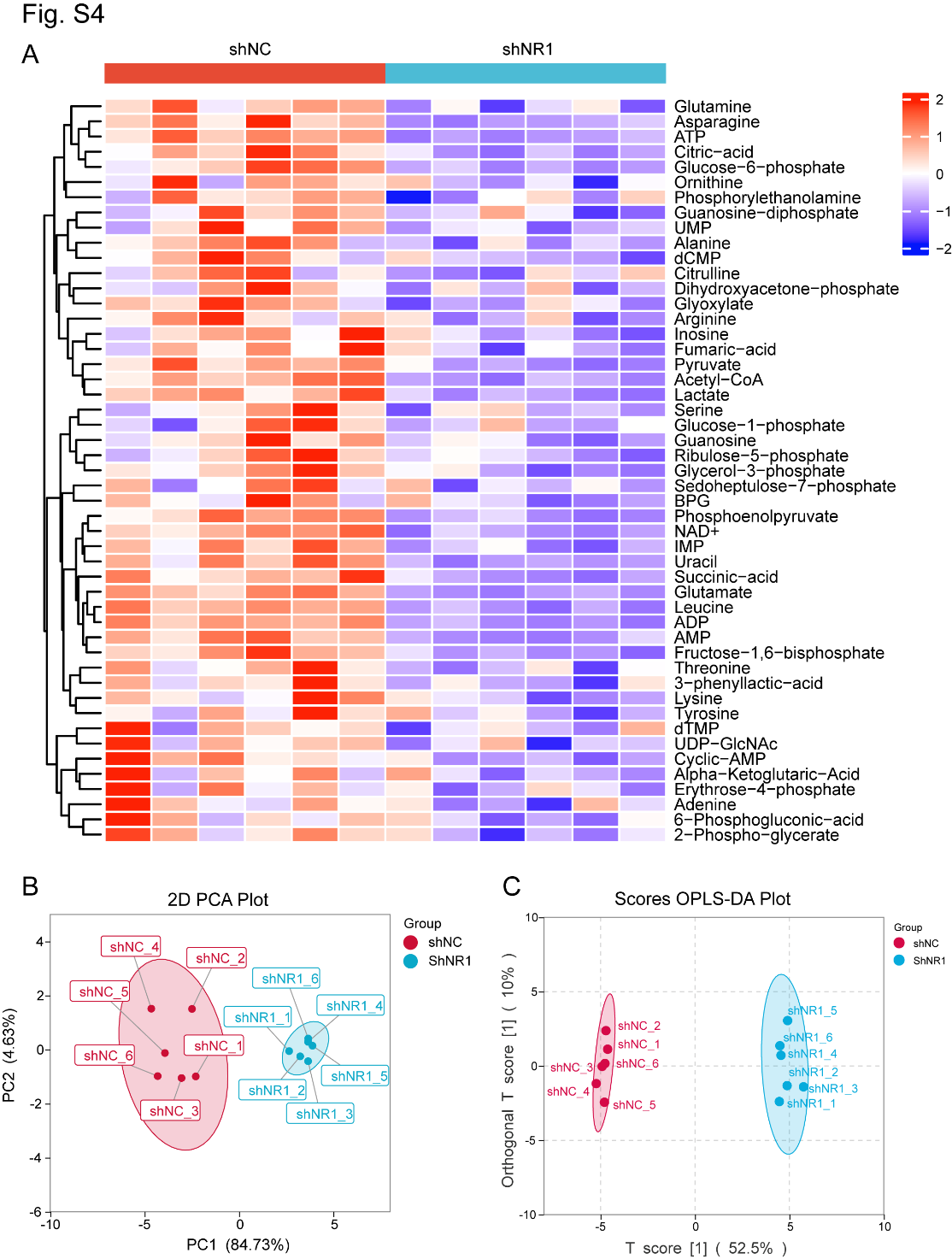


**Figure S4. Targeted energy metabolomics in SCs.**

(A). Hierarchical cluster analysis of energy-related metabolites detected in SCs. Hierarchical clustering analysis based on normalized peak intensities of intracellular metabolites. Rows indicate individual metabolites, while columns represent individual replicates, with a total of 6 replicates per group. For each metabolite, the colors represent relative metabolite abundance values normalized in the -2 (blue = low abundance) and 2 (red = high abundance) ranges. (B-C). Principal component analysis (PCA) (B) and partial least squares-discriminant analysis (OPLS-DA) (C) of metabolites revealed significant separation between the NR1-deficient group and the control group.


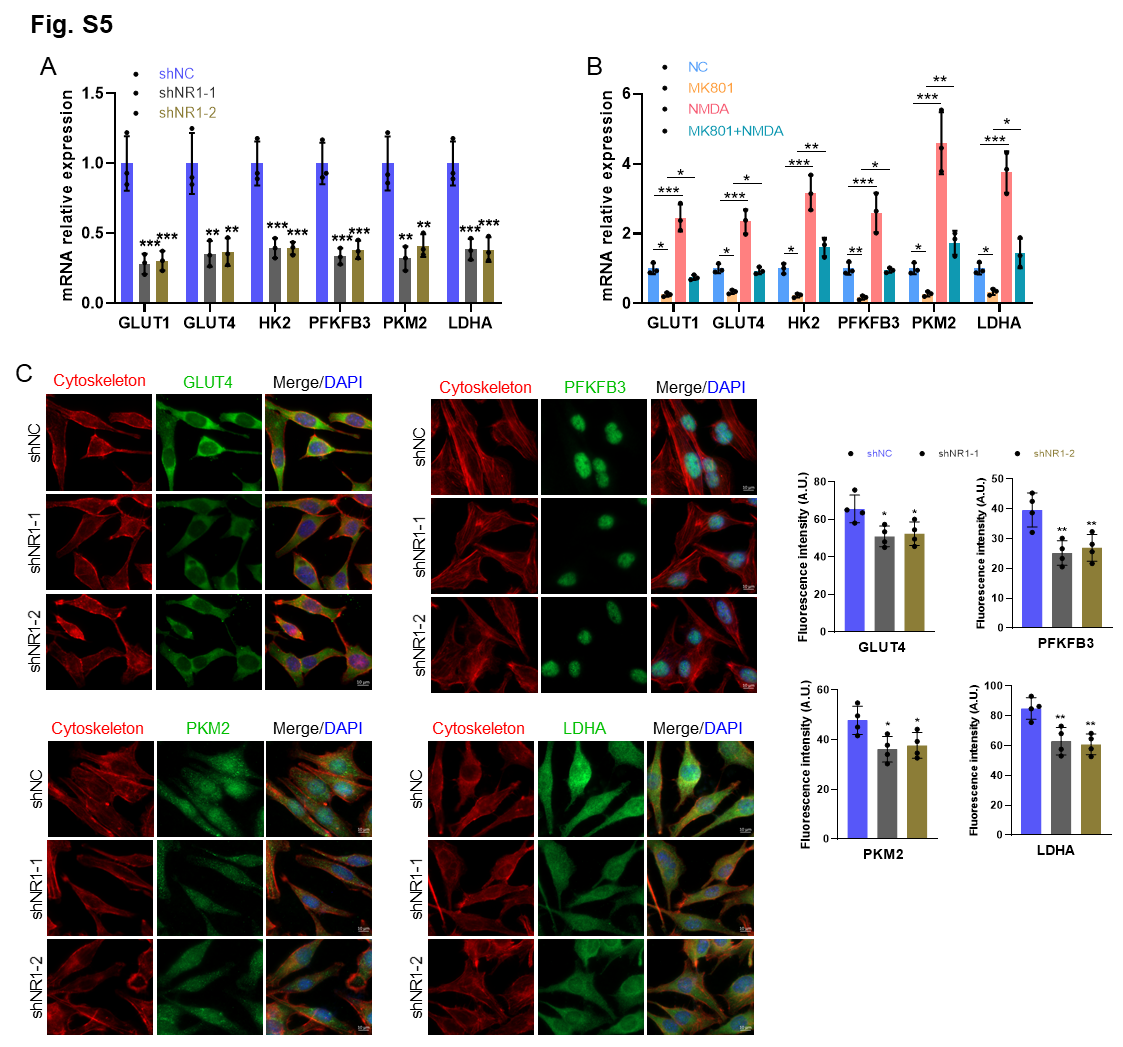


**Figure S5. Quantitative analysis of the expression of glycolysis-related genes in SCs.**

(A-B). qRT‒PCR analysis of the expression levels of glycolysis-related genes including GLUT1, GLUT4, HK2, PFKFB3, PKM2 and LDHA, in SCs. n=3. (C). Representative immunofluorescence and quantification of GLUT4, PFKFB3, PKM2 and LDHA in NR1-deficient SCs and control SCs. Scale bar, 10 μm. N = 4, n ≥ 10 fields/group. **p* < 0.05, ***p* < 0.01, ****p* < 0.001.

**
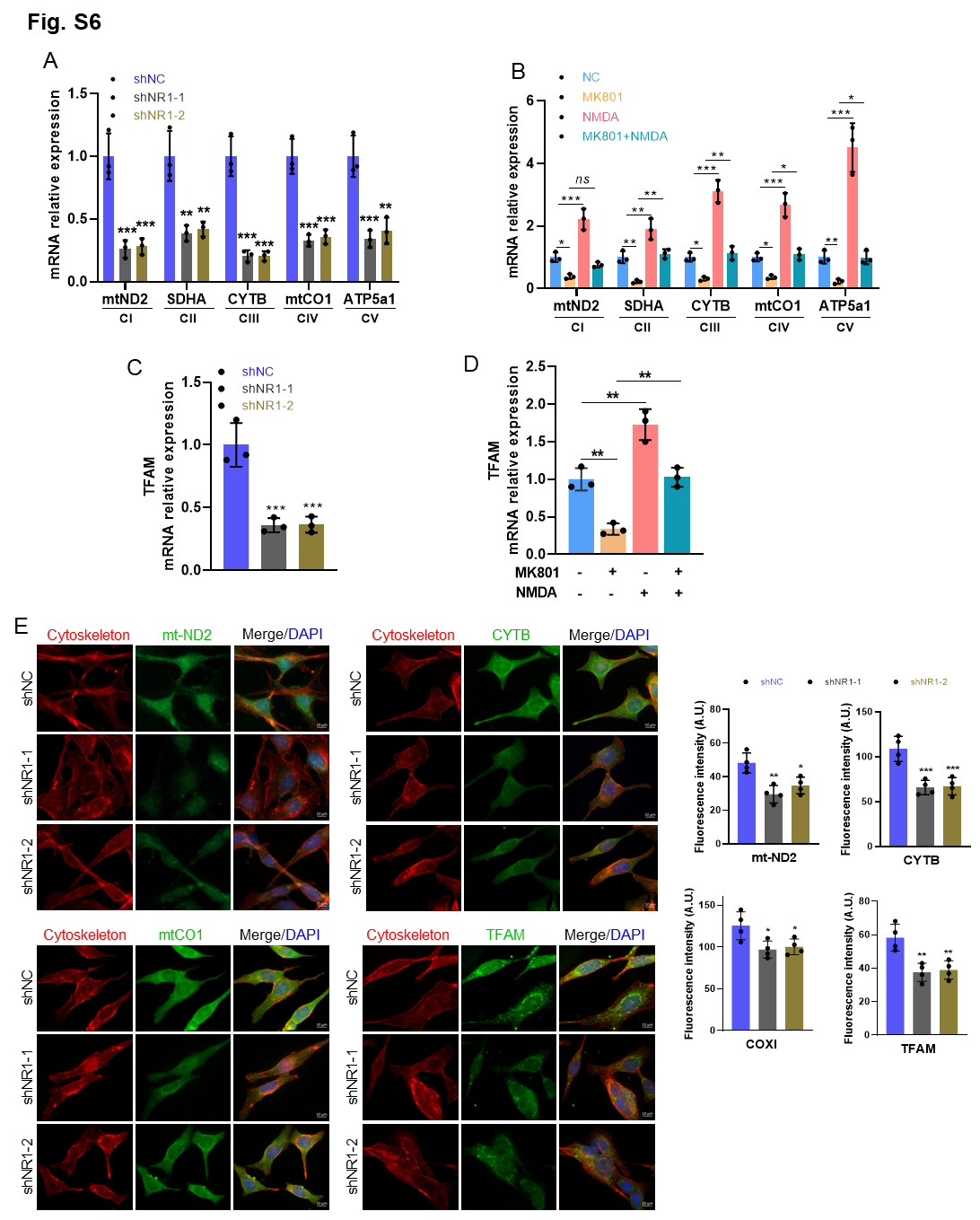
Figure S6. Quantitative analysis of the expression of mitochondrial respiration-related genes in SCs.**

(A-B). qRT‒PCR analysis of the gene expression levels of mitochondrial ETC components, including mt-ND2, SDHA, mt-CYTB, mt-CO1 and mt-ATP5a1, in SCs. n = 3. (C-D). qRT‒PCR analysis of the gene expression levels of TFAM in SCs. n = 3. (E). Representative immunofluorescence and quantification of mt-ND2, mt-CYTB, mt-CO1 and TFAM in NR1-deficient SCs and control SCs. Scale bar, 10 μm. N = 4, n ≥ 10 fields/group. *ns*, not significant, **p* < 0.05, ***p* < 0.01, ****p* < 0.001.

**
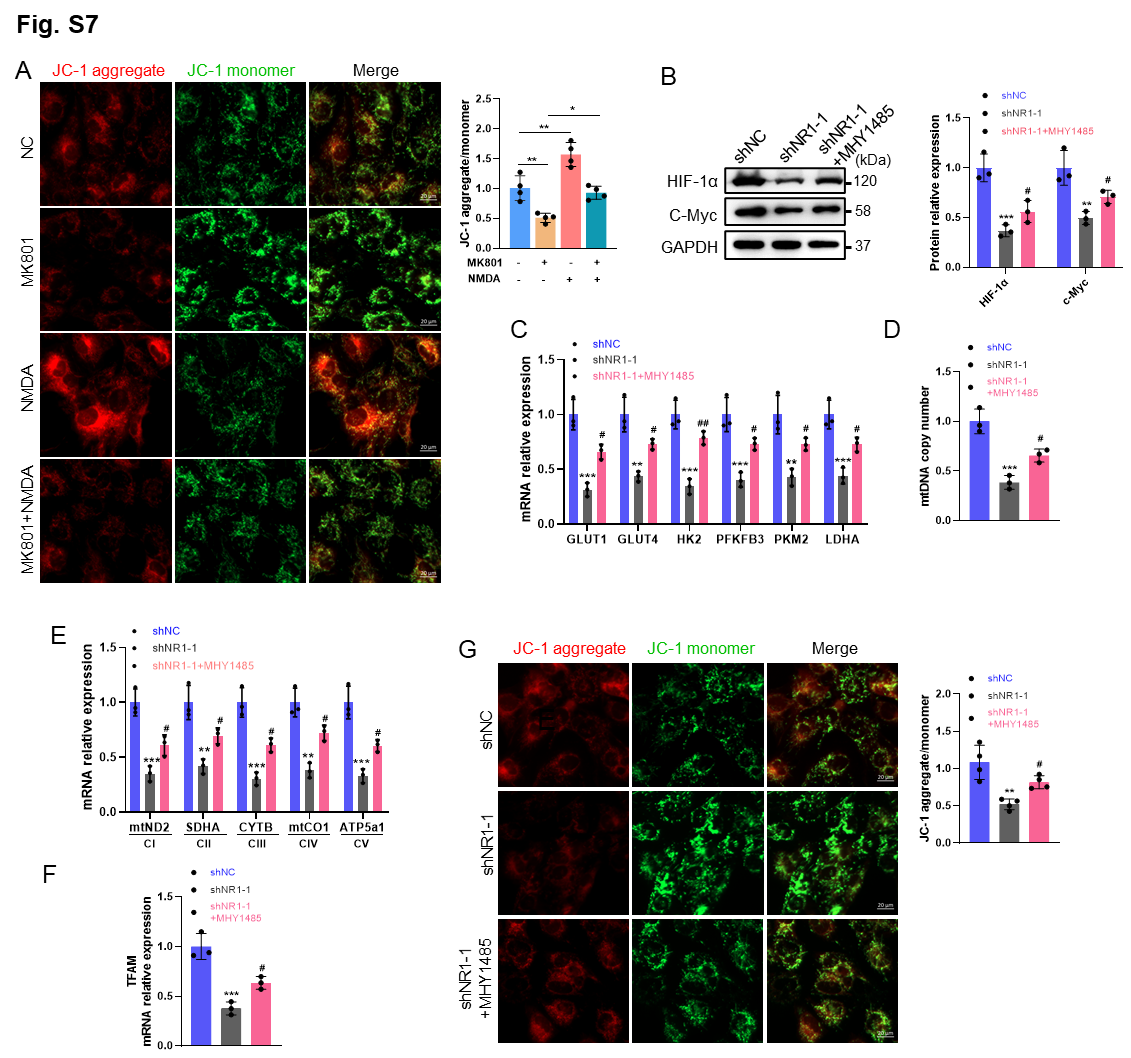
**

**Figure S7. mTOR activator rescues impaired glycolytic flux and oxidative metabolism in NR1-deficient SCs.**

(A). JC-1 probe staining and quantification of the mitochondrial membrane potential (MMP) in SCs treated with different drugs. Scale bars, 20 μm. N = 4, n ≥10 fields/group. **p* < 0.05, ***p* < 0.01. (B-F). NR1-deficient SCs (shNR1-1) were treated with the mTOR activator MHY1485 (5 μM) for 24 h. (B). The expression of HIF-1α and c-Myc was detected by western blot. n = 3. (C). qRT-PCR analysis of the gene expression of GLUT1, GLUT4, HK2, PFKFB3, PKM2 and LDHA in SCs. n = 3. (D). qRT‒PCR analysis of mtDNA copy number levels in SCs. n = 3. (E-F). qRT‒PCR analysis of the gene expression of mt-ND2, SDHA, mt-CYTB, mt-CO1, mt-Atp5a1 and TFAM in SCs. n = 3. (G). JC-1 probe was used to determine the MMP in SCs, and the JC-1 aggregate/monomer ratio was quantified. Scale bars, 20 μm. N = 4, n ≥ 10 fields/group. ***p* < 0.01, ****p* < 0.001, compared with shNC; ^#^*p* < 0.05, ^##^*p* < 0.01, compared with shNR1-1.

**
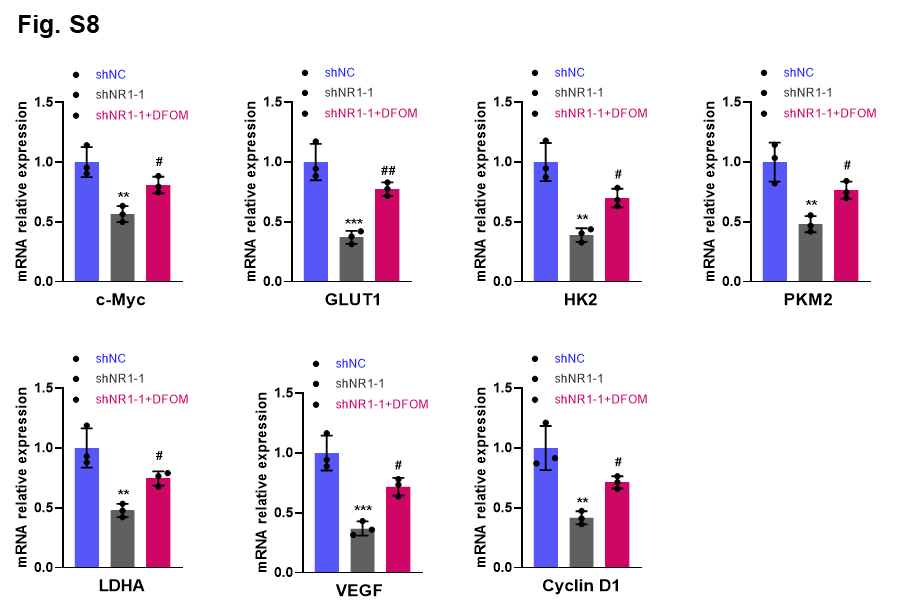
**

**Figure S8. HIF-1α activator rescues impaired glycolysis-related genes and cell growth-related genes in NR1-deficient SCs.**

NR1-deficient SCs (shNR1-1) were treated with the HIF-1α activator deferoxamine mesylate (DFOM, 10 μM) for 24 h. qRT‒PCR analysis of the gene expression of c-Myc, GLUT1, HK2, PKM2, LDHA, VEGF and Cyclin D1 in SCs. ***p* < 0.01, ****p* < 0.001, compared with shNC; ^#^*p* < 0.05, ^##^*p* < 0.01, compared with shNR1-1.
