# Supplementary material for: NMDA Receptors Coordinate Metabolic Reprogramming and Mitophagy in Schwann Cells to Promote Peripheral Nerve Regeneration
Source: Research (Wash D C). 2025 Aug 5;8:0825. doi: 10.34133/research.0825 (PMC12322490; doi:10.34133/research.0825)
Supplement: Supplementary 1 — Figs. S1 to S8 Tables S1 and S2 [file research.0825.f1.zip › Supplemental table.docx]

**Supplemental Table**

**Supplemental table 1. Primer sequences for qRT‒qPCR.**

| Gene |  | Primer sequence (5’-3’) |
| --- | --- | --- |
| NR1 | Forward | CTGCGACCCCAAGATTGTCAA |
|  | Reverse | TATTGGCCTGGTTTACTGCCT |
| NR2A | Forward | TGATGAACCGCACTGACCCTA |
|  | Reverse | GGAAGAACGTGGATGTCGGA |
| NR2B | Forward | GCCATGAACGAGACTGACCC |
|  | Reverse | GCTTCCTGGTCCGTGTCATC |
| NR2C | Forward | GGGATCTGCCATAACGAGAAG |
|  | Reverse | GCACTGAGTGTCGAAGTTTCCA |
| NR2D | Forward | GCCCATTCTCGACTTCCTGTC |
|  | Reverse | CCTTGGGTGTGAGTACGAGC |
| NR3A | Forward | CGGGAGAGTCAGAATCCCCT |
|  | Reverse | CCTGGCATAGCAACAAGCTAA |
| NR3B | Forward | TCTGGAGCTAGTGGCCGTC |
|  | Reverse | GCGCCTCGGGAAAGGTTATAG |
| GLUT1 | Forward | CAGTTCGGCTATAACACTGGTG |
|  | Reverse | GCCCCCGACAGAGAAGATG |
| GLUT4 | Forward | GGACCGGATTCCATCCCAC |
|  | Reverse | TCCCAACCATTGAGAAATGATGC |
| HK2 | Forward | ATGATCGCCTGCTTATTCACG |
|  | Reverse | CGCCTAGAAATCTCCAGAAGGG |
| PFKFB3 | Forward | CAACTCCCCAACCGTGATTGT |
|  | Reverse | GAGGTAGCGAGTCAGCTTCTT |
| PKM2 | Forward | GTGCCGCCTGGACATTGACTC |
|  | Reverse | ATTCAGCCGAGCCACATTCATCC |
| LDHA | Forward | TCGCACCTTGTAGCCGTTATTGG |
|  | Reverse | ACTGCCCTCCCGCTCTTCTC |
| COX2 | Forward | ATAACCGAGTCGTTCTGCCAAT |
|  | Reverse | TTTCAGAGCATTGGCCATAGAA |
| Rsp18 | Forward | GTGTTAGGGGACTGGTGGACA |
|  | Reverse | CATCACCCACTTACCCCCAAA |
| mt-ND2 | Forward | ACCAAATCTCTCCCTCACTAAACG |
|  | Reverse | CCACCTCAACTGCCTGCTATG |
| SDHA | Forward | TTACAAAGTGCGGGTCGATGA |
|  | Reverse | TGTTCCCCAAACGGCTTCTT |
| mt-CYTB | Forward | CCCACCCCATATTAAACCCG |
|  | Reverse | GAGGTATGAAGGAAAGGTATAAGGG |
| mt-CO1 | Forward | TCCCAGATATAGCATTCCCACG |
|  | Reverse | ACTGTTCATCCTGTTCCTGC |
| mt-ATP5a1 | Forward | TCTCCATGCCTCTAACACTCG |
|  | Reverse | CCAGGTCAACAGACGTGTCAG |
| TFAM | Forward | ATTCCGAAGTGTTTTTCCAGCA |
|  | Reverse | TCTGAAAGTTTTGCATCTGGGT |
| c-Myc | Forward | ATGCCCCTCAACGTGAACTTC |
|  | Reverse | CGCAACATAGGATGGAGAGCA |
| VEFG | Forward | CTGCCGTCCGATTGAGACC |
|  | Reverse | CCCCTCCTTGTACCACTGTC |
| Cyclin D1 | Forward | GCGTACCCTGACACCAATCTC |
|  | Reverse | CTCCTCTTCGCACTTCTGCTC |
| GAPDH | Forward | AACGACCCCTTCATTGAC |
|  | Reverse | TCCACGACATACTCAGCAC |

**Supplemental table 2. Details of primary and secondary antibodies**

| Antibodies | Cat. number | Applications | Source |
| --- | --- | --- | --- |
| Rabbit anti-NR1 | 32-0500 | WB: 1:500  IF: 1:100 | Invitrogen |
| Rabbit anti-NR2C | PA5-41001 | IF: 1:100 | Invitrogen |
| Rabbit anti-NR2D | PA5-101608 | IF: 1:100 | Invitrogen |
| Mouse anti-S100β | MA5-12969 | IF: 1:200 | Invitrogen |
| Rabbit anti-NF200 | ab8135 | IF: 1:200 | Abcam |
| Rat anti-MBP | MAB386 | IF: 1:200 | Sigma |
| Rabbit anti-NGFR | PA5-27656 | IF: 1:200 | Invitrogen |
| Mouse anti-TuJ1 | MA1-118 | IF: 1:500 | Invitrogen |
| Rabbit anti-GLUT1 | ab115730 | WB: 1:500 IF: 1:200 | Abcam |
| Mouse anti-GLUT4 | 66846-1-Ig | WB: 1:1000 IF: 1:250 | Proteintech |
| Rabbit anti-PFKFB3 | ab181861 | WB: 1:1000 IF: 1:200 | Abcam |
| Rabbit anti-LDHA | DF6280 | WB: 1:1000 IF: 1:200 | Affinity |
| Rabbit anti-HK2 | DF6176 | WB: 1:2000 IF: 1:400 | Affinity |
| Rabbit anti-PKM2 | AF5234 | WB: 1:1000 IF: 1:200 | Affinity |
| Rabbit anti-mt-ND2 | A17968 | IF: 1:100 | ABclonal |
| Rabbit anti-mt-ND2 | 19704-1-AP | WB: 1:500 | Proteintech |
| Rabbit anti-SDHA | 14865-1-AP | WB: 1:1000 IF: 1:200 | Proteintech |
| Rabbit anti-mt-CYTB | 55090-1-AP | WB: 1:1000 IF: 1:200 | Proteintech |
| Mouse anti-mt-CO1 | 459600 | WB: 1:500 IF: 1:200 | Invitrogen |
| Rabbit anti-mt-ATP5a1 | 14676-1-AP | WB: 1:2000 IF: 1:500 | Proteintech |
| Rabbit anti-TFAM | 22586-1-AP | WB: 1:5000 IF: 1:500 | Proteintech |
| Rabbit anti-MCT1 | 20139-1-AP | WB: 1:1000 IF: 1:200 | Proteintech |
| Rabbit anti-MFN2 | 12186-1-AP | WB: 1:1000 | Proteintech |
| Rabbit anti-OPA1 | 27733-1-AP | WB: 1:1000 | Proteintech |
| Rabbit anti-DRP1 (C-terminal) | 12957-1-AP | WB: 1:1000 | Proteintech |
| Rabbit anti-FIS1 | 10956-1-AP | WB: 1:1000 | Proteintech |
| Rabbit anti-AMPKα1+ AMPKα2 | ab207442 | WB: 1:1000 | Abcam |
| Rabbit anti-p- AMPKα1(Thr183) + AMPKα2(Thr172) | ab133448 | WB: 1:1000 | Abcam |
| Rabbit anti-PI3K p85α | AF6241 | WB: 1:1000 | Affinity |
| Rabbit anti-p-PI3K p85α (Tyr607) | AF3241 | WB: 1:500 | Affinity |
| Rabbit anti-AKT | AF6261 | WB: 1:1000 | Affinity |
| Rabbit anti-p-AKT (Ser473) | AF0016 | WB: 1:500 | Affinity |
| Rabbit anti-mTOR | 2983 | WB: 1:1000 | Cell Signal Technology |
| Rabbit anti-p-mTOR (Ser2448) | 5536 | WB: 1:1000 | Cell Signal Technology |
| Rabbit anti-p70S6K | AF6226 | WB: 1:1000 | Affinity |
| Rabbit anti-p-p70S6K (Thr389/Thr412) | AF3228 | WB: 1:500 | Affinity |
| Rabbit anti-HIF-1α | 20960-1-AP | WB: 1:2000 | Proteintech |
| Rabbit anti-c-Myc | 10828-1-AP | WB: 1:2000 | Proteintech |
| Rabbit anti-STAT3 | 12640 | WB: 1:1000 | Cell Signal Technology |
| Rabbit anti-p-STAT3(Tyr705) | 9145 | WB: 1:1000 | Cell Signal Technology |
| Rabbit anti-Atg13 | 13273 | WB: 1:1000  IP: 1:50 | Cell Signal Technology |
| Rabbit anti-ATR | 13934 | WB: 1:1000  IP: 1:100 | Cell Signal Technology |
| Rabbit anti-Parkin | AF0235 | WB: 1:500 | Affinity |
| Mouse anti-PINK1 | sc-517353 | WB: 1:200 | Santa Cruz |
| Rabbit anti-LC3B | 83506 | WB: 1:1000 IF: 1:200 | Cell Signal Technology |
| Mouse anti-GAPDH | AC033 | WB: 1:10000 | ABclonal |
| Goat anti-mouse IgG-HRP | AS064 | WB: 1:5000 | ABclonal |
| Goat anti-Rabbit IgG-HRP | AS063 | WB: 1:5000 | ABclonal |
| Goat anti-mouse IgG, Alexa Fluor™ 488 | A32723 | IF: 1:500 | Invitrogen |
| Donkey anti-Mouse IgG, Alexa Fluor™ 555 | A31570 | IF: 1:500 | Invitrogen |
| Goat anti-mouse IgG2a, Alexa Fluor™ 555 | A21137 | IF: 1:500 | Invitrogen |
| Goat anti-rabbit IgG, Alexa Fluor™ 488 | A-11034 | IF: 1:500 | Invitrogen |
| Donkey Anti-Rat IgG, Alexa Fluor™ 555 | ab150154 | IF: 1:500 | Abcam |
